# The Indecision Model of Psychophysical Performance in Dual-Presentation Tasks: Parameter Estimation and Comparative Analysis of Response Formats

**DOI:** 10.3389/fpsyg.2017.01142

**Published:** 2017-07-12

**Authors:** Miguel A. García-Pérez, Rocío Alcalá-Quintana

**Affiliations:** Departamento de Metodología, Facultad de Psicología, Universidad Complutense Madrid, Spain

**Keywords:** indecision model, response format, psychometric function, psychophysical function, parameter estimation, goodness of fit

## Abstract

Psychophysical data from dual-presentation tasks are often collected with the two-alternative forced-choice (2AFC) response format, asking observers to guess when uncertain. For an analytical description of performance, psychometric functions are then fitted to data aggregated across the two orders/positions in which stimuli were presented. Yet, order effects make aggregated data uninterpretable, and the bias with which observers guess when uncertain precludes separating sensory from decisional components of performance. A ternary response format in which observers are also allowed to report indecision should fix these problems, but a comparative analysis with the 2AFC format has never been conducted. In addition, fitting ternary data separated by presentation order poses serious challenges. To address these issues, we extended the indecision model of psychophysical performance to accommodate the ternary, 2AFC, and same–different response formats in detection and discrimination tasks. Relevant issues for parameter estimation are also discussed along with simulation results that document the superiority of the ternary format. These advantages are demonstrated by fitting the indecision model to published detection and discrimination data collected with the ternary, 2AFC, or same–different formats, which had been analyzed differently in the sources. These examples also show that 2AFC data are unsuitable for testing certain types of hypotheses. matlab and R routines written for our purposes are available as Supplementary Material, which should help spread the use of the ternary format for dependable collection and interpretation of psychophysical data.

Psychophysical data are widely collected with dual-presentation (2P) tasks whose trials display two stimuli of selected magnitudes. These tasks are often administered with the two-alternative forced-choice (2AFC) response format in which observers report the stimulus perceived to have some characteristic. Thus, in 2P *detection* tasks, one stimulus (the standard) has null magnitude on all trials whereas the other (the test) has a non-null magnitude that varies across trials and observers report which presentation displayed the non-null stimulus. In 2P *discrimination* tasks, the standard has a fixed non-null magnitude whereas the test varies in magnitude across trials and observers report which presentation displayed a stimulus of, say, higher magnitude. Presentations can occur in consecutive temporal intervals or in adjacent spatial positions, rendering temporal or spatial 2P tasks. Because the temporal or spatial aspect is formally inconsequential (though perceptually relevant; see García-Pérez et al., 2005), presentations will here be denoted “first” and “second” to indicate either temporal order or positional order (location).

Across trials, standard and test are displayed about equally often in each presentation order except under the *reminder paradigm* (Macmillan and Creelman, 2005, p. 180–182) in which the standard is presented first on all trials, but this paradigm will not be considered here. Responses are aggregated across presentation orders and binned by test magnitude (henceforth, level) to compute the proportion of trials in which observers were correct in detection tasks or in which they reported the test to be subjectively higher in discrimination tasks (i.e., they chose the first presentation when the test was first or the second presentation when it was second). A plot of these proportions as a function of test level delineates a curve to which a psychometric function is fitted for an analytical description of performance.

Two aspects of this widespread practice are questionable. One of them is the aggregation of responses across presentation orders, which is justifiable only if performance is invariant with presentation order. Overwhelming evidence to the contrary has been reported in a number of sensory modalities and stimulus dimensions (see, e.g., Jamieson and Petrusic, 1975; Allan, 1977; Jamieson, 1977; Masin and Agostini, 1991a,b; Hellström, 2003; Hellström and Rammsayer, 2004, 2015; Alcalá-Quintana and García-Pérez, 2011; García-Pérez and Alcalá-Quintana, 2011a; Dyjas et al., 2012; Dyjas and Ulrich, 2014; van den Berg et al., 2017). This evidence led Ulrich and Vorberg (2009; see also García-Pérez and Alcalá-Quintana, 2011b) to stress that separate psychometric functions should be fitted for each presentation order under suitable constraints and to develop software that accomplishes this goal, although only for discrimination tasks (Bausenhart et al., 2012).

The second questionable aspect is the assumption that observers can always make an informed decision about which stimulus has the target characteristic. The assumption seems grounded on the feasibility of a decision based on the perceived difference relative to a fixed cut point (typically placed at 0), as posited by the signal-detection-theoretic difference model for 2AFC responding (see Figure 7.2 in Macmillan and Creelman, 2005). However, such decision model is in contradiction with the difference model for same–different responding, a response format for 2P tasks in which observers report instead whether or not the two stimuli are subjectively equal. The decision model here posits that observers cannot tell which stimulus has a higher magnitude (and, hence, respond “same”) if the perceived difference is within some vicinity of 0 (see Figure 9.5 in Macmillan and Creelman, 2005). In other words, the decision rule presumed to underlie performance under 2AFC responding (referred to as the *comparative* task; Schneider, 2006; Dyjas and Ulrich, 2014) implies that observers will never report equality under same–different responding (referred to as the *equality* task), whereas the decision rule presumed to underlie performance in the equality task implies that observers must guess in a comparative task when both stimuli are subjectively equal. This contradiction may be explained away with the *ad-hoc* argument that observers are capable of perceiving subjective equality only under same–different responding. However, researchers acknowledge that observers may also perceive equality under 2AFC responding and explicitly instruct them to guess in such cases (e.g., Allan, 1977; Tolhurst and Barfield, 1978; Jenkins, 1985; Schneider, 2006; Norman et al., 2011; Brown et al., 2015). It would certainly make more sense to ask observers to report their indecision instead. Indeed, guessing alters psychometric functions according to the bias with which observers respond “first” or “second” when uncertain (see, e.g., Figure 1 in Pastore and Farrington, 1996) and introduces a contamination that precludes separating the sensory and decisional components of performance.

Removing this contamination requires administering the 2P task with a ternary response format in which observers are still given the classical response options (i.e., choose one stimulus or the other) but they are also allowed to report that both stimuli were subjectively equal. The ternary format was widely used by Fechner (1860/1966) and by most of the early psychophysicists (see the first few chapters in Link, 1992), but it fell in disuse when signal detection theory was introduced. A recent attempt to reinstate the ternary format (Rammsayer and Ulrich, 2001) did not meet immediate recognition perhaps because analyzing ternary data is not straightforward, less so when order effects have to be taken into account. In addition, it has never been established that the ternary format pays off: A comparison with binary response formats has never been conducted.

This paper has two goals. Firstly, to document the advantages of the ternary format relative to the 2AFC or same–different formats, in terms of the accuracy with which model parameters and performance measures can be estimated. Secondly, to discuss aspects of the fitting of psychometric functions to ternary detection and discrimination data, showing along the way that 2AFC data are unsuitable to test certain types of hypotheses. The indecision model (García-Pérez and Alcalá-Quintana, 2010a) is amended and extended for these purposes so that it also accommodates the 2AFC and same–different formats. A description of the amended model is first given, followed by a description of its application to binary response formats. Simulation results are then presented that document the higher accuracy of parameter estimates from ternary data. Aspects of parameter estimation and hypothesis testing are finally illustrated via analysis of published detection and discrimination data collected with the ternary, 2AFC, or same–different formats, which had been analyzed differently in the sources. Routines (in matlab and R) to fit the indecision model were written for our purposes and are available as Supplementary Material, which should help to spread the use of the ternary format. In empirical practice, use of the ternary response format only requires that observers be given a third response key to express indecision whenever needed, with no change in any other aspect of the psychophysical paradigm. Data analysis is subsequently adapted to the characteristics of ternary data but the routines just mentioned carry out that task.

## The ternary indecision model of psychophysical judgments

The original form of the indecision model has been presented elsewhere (García-Pérez and Alcalá-Quintana, 2010a,b, 2011a, 2013; García-Pérez, 2014a; Sridharan et al., 2014; see also García-Pérez and Peli, 2014, 2015; Pritchett and Murray, 2015; Self et al., 2015). The following description expands the model in some respects and highlights important features that are relevant to parameter estimation.

**Figure 1:**
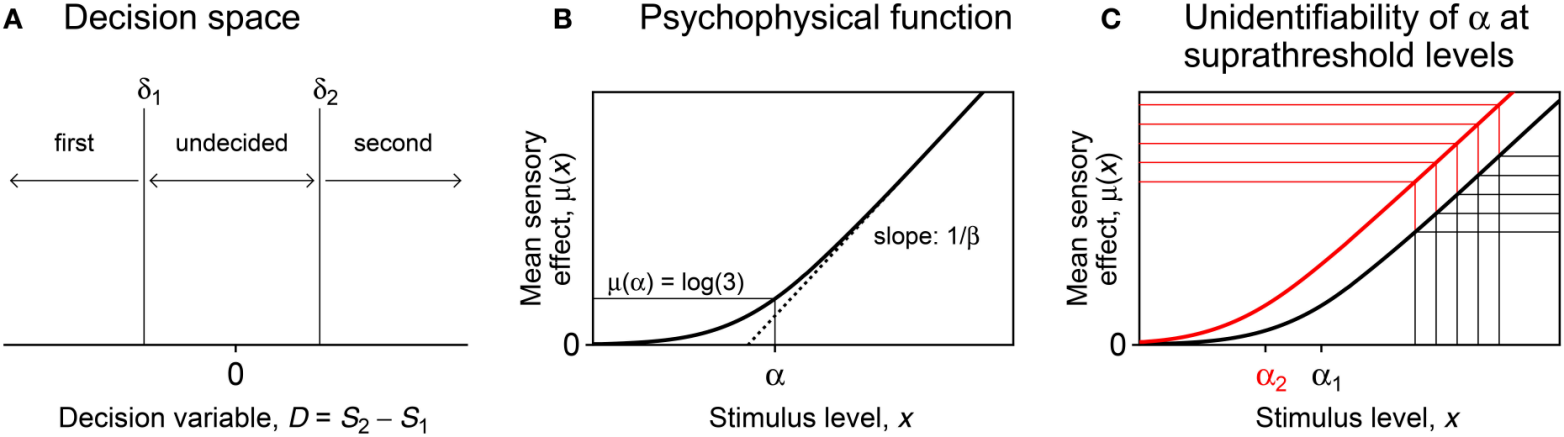
Components of the indecision model. **(A)** Decision space with boundaries at δ_1_ and δ_2_, not necessarily placed symmetrically about the null value of the decision variable *D* defined as the difference between the sensory effect *S*_2_ of the stimulus presented second and the sensory effect *S*_1_ of the stimulus presented first. The observer chooses the first presentation if *D* < δ_1_, chooses the second presentation if *D* > δ_2_, and is undecided if δ_1_ < *D* < δ_2_. **(B)** Psychophysical function μ in Equation (2) (solid curve) and its oblique asymptote (dotted line). **(C)** Illustration of the unidentifiability of parameter α in discrimination tasks at suprathreshold stimulus levels within the linear range of μ. Consider the five test levels indicated along the horizontal axis, with the standard stimulus at the central level. Whether mapped onto the subjective axis via the black curve or via the red curve (which differ only as to parameter α), the relative distance between the sensory effects of all pairs of stimuli are identical and only their locations along the vertical axis (which is immaterial) varies with α.

The indecision model is analogous to the signal-detection model for a rating task with three response categories. Thus, the decision variable *D* = *S*_2_ – *S*_1_ is the difference between the sensory effect *S*_2_ of the stimulus presented second and the sensory effect *S*_1_ of the stimulus presented first, and the decision space is partitioned into three regions each associated with one of the judgments (Figure 1A). Sensory effects are assumed to be normally distributed with unit variance and a mean determined by stimulus level, but normality can be replaced with distributional forms that are more appropriate in some cases (see, e.g., García-Pérez and Peli, 2014). The sensory effect *S* of a stimulus with level *x* is thus a random variable with density

(1) $$\begin{array}{l} f(s;x)=\frac{\text{1}}{\sqrt{\text{2}\pi }}\text{ exp }[-\frac{{\left(s\;-\;\mu \left(x\right)\right)}^{\text{2}}}{\text{2}}], \end{array}$$

where μ is the *psychophysical function* relating mean sensory effect to stimulus level.

The form of μ has been under scrutiny for decades and it is still unclear whether a unique form exists (Kornbrot, 2016). Nevertheless, some aspects of the mathematical form of μ are immaterial in 2P tasks, where the values μ(*x*_1_) and μ(*x*_2_) at the stimulus levels *x*_1_ and *x*_2_ displayed first and second are not crucial and only their difference matters. We use the form

(2) $$\begin{array}{l} \mu \left(x\right)=\log\left(1+2{\text{e}}^{\left(x-\alpha \right)/\beta }\right), \end{array}$$

an increasing function (Figure 1B) with a lower asymptote at *y* = 0 and an oblique asymptote at *y* = log(2) + (*x* − α)/β. Thus, mean sensory effects are null at low (imperceptible) levels, subsequently grow slowly and non-linearly, and finally grow linearly with a slope of 1/β in the suprathreshold range. This choice accommodates stimulus dimensions in any range and scale, but two considerations should be made.

In discrimination tasks, the standard may differ from the test along dimensions other than that of comparison (e.g., the dimension of comparison is line length but standard and test lines differ in orientation). When the extra dimension affects perceived magnitude, separate functions μ_s_ and μ_t_ hold for standard and test, and both must be considered. When test and standard differ only along the dimension of comparison or when the extra dimension in which they differ does not have perceptual effects, μ_s_ = μ_t_. This is also the case in detection tasks. The model is described here with μ_s_ ≠ μ_t_ because μ_s_ = μ_t_ results in straightforward simplifications. It should be stressed that, in some discrimination studies, whether or not μ_s_ = μ_t_ is a hypothesis that the data should allow testing.

The second consideration relates to the identifiability of the parameters of μ. In detection tasks, where the null standard at *x*_s_ sets an anchor at μ_s_(*x*_s_) = 0 along the subjective axis, test levels probe the initial non-linear range of μ_t_. Hence, parameters α_t_ and β_t_ are identifiable. In a suprathreshold discrimination task, where only the linear range of μ_t_ is involved, the anchor μ_s_(*x*_s_) ≠ 0 set by the standard is unknown and, given that μ_t_(*x*) − μ_s_(*x*_s_) = (*x* − α_t_ − β_t_(*x*_s_ − α_s_)/β_s_)/β_t_ within the linear range, parameter α_t_ is not identifiable. Figure 1C illustrates the unidentifiability of α_t_ when μ_s_ = μ_t_, which simplifies the preceding expression to μ_t_(*x*) − μ_s_(*x*_s_) = (*x* − *x*_s_)/β_t_ and makes even more explicit that α_t_ is not identifiable. This unidentifiability is an inherent feature of difference models and it has been shown to arise for many forms of μ (e.g., García-Pérez and Alcalá-Quintana, 2013; García-Pérez, 2014a), but it is inconsequential when the non-identifiable parameters are replaced with identifiable combinations or when one or more of them are fixed to arbitrary but reasonable anchor values.

**Figure 2:**
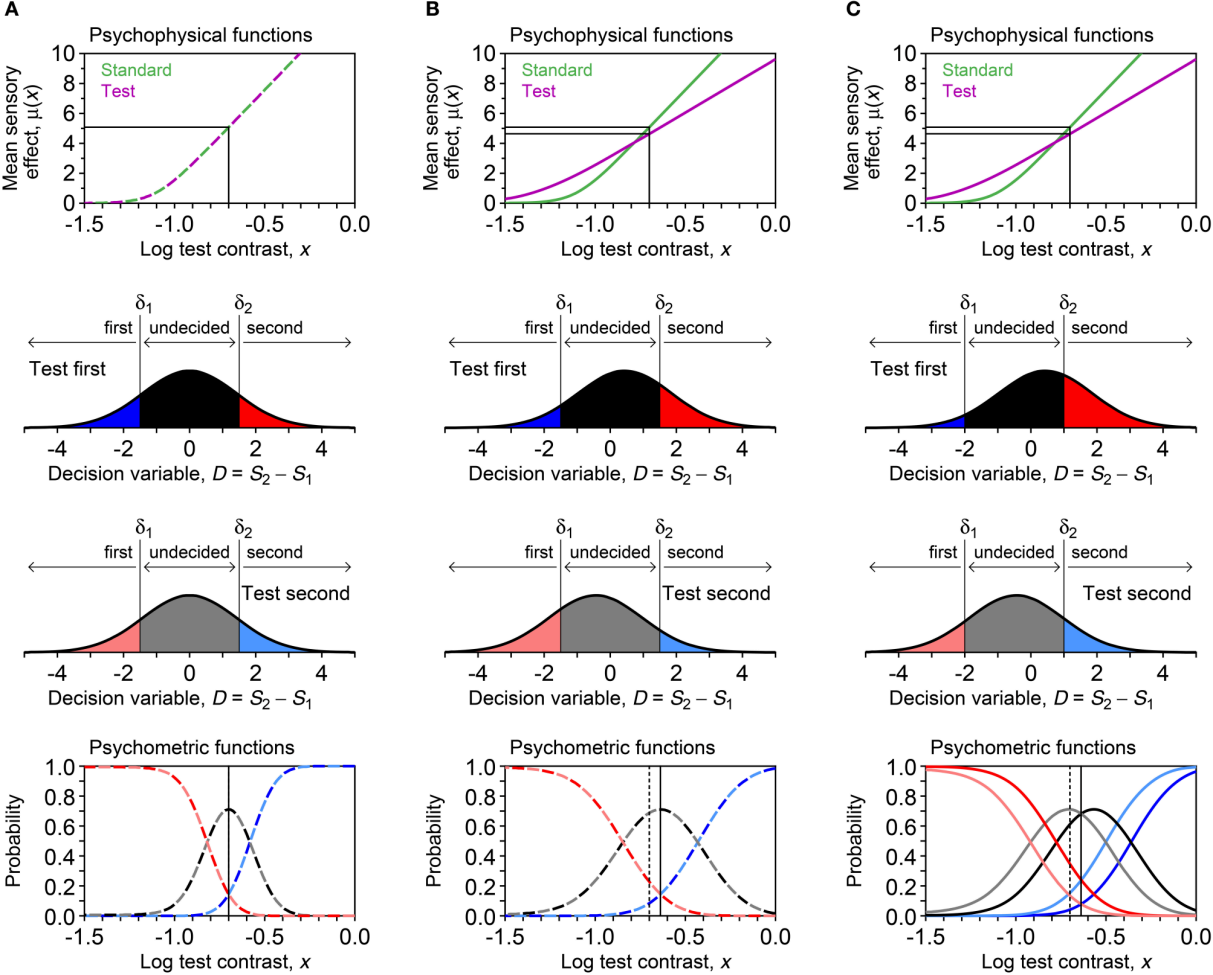
Illustration of the model under three scenarios (columns) in a visual contrast discrimination task: **(A)** μ_t_ = μ_s_ and δ_1_ = −δ_2_; **(B)** μ_t_ ≠ μ_s_ and δ_1_ = −δ_2_; **(A)** μ_t_ ≠ μ_s_ and δ_1_ ≠ −δ_2_. First row: Assumed psychophysical functions for standard and test stimuli, given by Equation (2) with α_s_ = α_t_ = −1.05 and β_s_ = β_t_ = 0.08 in **(A)** or α_s_ = −1.05, α_t_ = −1.25, β_s_ = 0.08, and β_t_ = 0.14 in **(B)** and **(C)**. The standard level (*x*_s_ = −0.7) and its mapping onto subjective space via either psychophysical function are indicated by the vertical–horizontal thin line segments. Second row: Decision space and distribution of the decision variable *D* when a test stimulus at *x* = *x*_s_ is presented first. The shaded areas give the probability of each possible judgment, determined by decision boundaries at δ_1_ = −1.5 and δ_2_ = 1.5 in **(A)** and **(B)** or at δ_1_ = −2 and δ_2_ = 1 in **(C)**. Third row: Analogous to the second row, but the distribution of *D* is shown when the test is presented second. Fourth row: Psychometric functions for each possible response under each presentation order, with color codes as in the second and third rows. The dashed vertical line indicates the standard level; the solid vertical line, which occludes the dashed vertical line in **(A)**, indicates the PSE.

The decision variable *D* is normally distributed with variance 2 and mean μ_s_(*x*_s_) − μ_t_(*x*) if the test is presented first or μ_t_(*x*) − μ_s_(*x*_s_) if the test is presented second. Given a decision space with boundaries at δ_1_ and δ_2_ (Figure 1A), the probabilities *p*_F,*m*_, *p*_U,*m*_, and *p*_S,*m*_ of a “first” (F), “undecided” (U), or “second” (S) judgment when the test at level *x* is presented in interval *m* ∈ {1, 2} are

(3a) $$\begin{array}{l} p_{\text{F},1}\left(x\right)=\Phi \left(\frac{\delta_{\text{1}}-\mu_{\text{s}}\left(x_{\text{s}}\right)+\mu_{\text{t}}\left(x\right)}{\sqrt{2}}\right) \end{array}$$

(3b) $$\begin{array}{l} p_{\text{U},1}\left(x\right)=\Phi \left(\frac{\delta_{2}-\mu_{\text{s}}\left(x_{\text{s}}\right)+\mu_{\text{t}}\left(x\right)}{\sqrt{2}}\right)\\ -\;\Phi \left(\frac{\delta_{\text{1}}-\mu_{\text{s}}\left(x_{\text{s}}\right)+\mu_{\text{t}}\left(x\right)}{\sqrt{2}}\right) \end{array}$$

(3c) $$\begin{array}{l} p_{S,1}\left(x\right)=1-\Phi \left(\frac{\delta_{2}-\mu_{\text{s}}\left(x_{\text{s}}\right)+\mu_{\text{t}}\left(x\right)}{\sqrt{2}}\right) \end{array}$$

(3d) $$\begin{array}{l} p_{\text{F},2}\left(x\right)=\Phi \left(\frac{\delta_{\text{1}}-\mu_{\text{t}}\left(x\right)+\mu_{\text{s}}\left(x_{\text{s}}\right)}{\sqrt{2}}\right) \end{array}$$

(3e) $$\begin{array}{l} p_{U,2}\left(x\right)=\Phi \left(\frac{\delta_{2}-\mu_{\text{t}}\left(x\right)+\mu_{\text{s}}\left(x_{\text{s}}\right)}{\sqrt{2}}\right)\\ -\;\Phi \left(\frac{\delta_{\text{1}}-\mu_{\text{t}}\left(x\right)+\mu_{\text{s}}\left(x_{\text{s}}\right)}{\sqrt{2}}\right) \end{array}$$

(3f) $$\begin{array}{l} p_{S,2}\left(x\right)=1-\Phi \left(\frac{\delta_{2}-\mu_{\text{t}}\left(x\right)+\mu_{\text{s}}\left(x_{\text{s}}\right)}{\sqrt{2}}\right), \end{array}$$

where Φ is the unit-normal cumulative distribution. Figure 2 illustrates the model for a discrimination task in three scenarios: μ_s_ = μ_t_ with δ_1_ = −δ_2_ (Figure 2A), μ_s_ ≠ μ_t_ with δ_1_ = −δ_2_ (Figure 2B), and μ_s_ ≠ μ_t_ with δ_1_ ≠ −δ_2_ (Figure 2C). If δ_1_ and δ_2_ are not placed symmetrically about *D* = 0 (i.e., δ_1_ ≠ −δ_2_), *decisional bias* occurs and the psychometric functions for each presentation order are displaced in opposite directions relative to the common location that they would have without such bias (compare the bottom panels in Figures 2B,C). With or without decisional bias, μ_s_ ≠ μ_t_ shifts the vertical axis of symmetry away from *x* = *x*_s_ (compare the bottom panels in Figures 2A,B).

The *point of subjective equality* (PSE) is the test level at which the (average) perceived magnitudes of test and standard are equal. Under 2AFC responding, the PSE is extracted as the abscissa at which the psychometric function for “test higher” responses evaluates to 0.5, but this method is inappropriate under the ternary format. By definition, the PSE is the level *x*_PSE_ at which μ_t_(*x*_PSE_) = μ_s_(*x*_s_) and, thus, $x_{\text{PSE}}=\mu_{\text{t}}^{-1}\left(\mu_{\text{s}}\left(x_{\text{s}}\right)\right)$. The PSE is then extracted from the estimated psychophysical functions and, naturally, *x*_PSE_ = *x*_s_ when μ_t_ = μ_s_.

The *difference limen* (DL) can also be determined from discrimination data collected with the ternary format. Under 2AFC responding, the DL is extracted as the distance between the PSE and the level *x*_DL_ at which the psychometric function for “test higher” responses evaluates to, say, 0.75, but this approach is again inappropriate under the ternary format. By definition, *x*_DL_ is the level at which the probability is 0.75 that the sensory effect *S*_t_ of the test exceeds the sensory effect *S*_s_ of the standard, that is, the solution of Prob(*S*_t_ − S_s_ > 0) =.75. With normally-distributed sensory effects, $x_{\text{DL}}=\mu_{\text{t}}^{-1}\left(\mu_{\text{s}}\left(x_{\text{s}}\right)+z_{0.75}\sqrt{2}\right)$, where *z*_0.75_ is the 75th quantile of the unit-normal distribution.

Similar considerations hold for detection tasks in which PSEs and DLs are undefined. Instead, the *detection threshold* is defined under 2AFC responding as the level at which the psychometric function for correct responses evaluates to, say, 0.84. This method is inappropriate under the ternary format. The detection threshold is the level θ at which the probability is 0.84 that the sensory effect of the test exceeds that of the null standard, that is, the solution of Prob(*S*_t_ − S_s_ > 0) =.84. With normally-distributed sensory effects, $\theta =\mu_{\text{t}}^{-1}\left(z_{0.84}\sqrt{2}\right)$. For a thorough discussion of detection and discrimination thresholds and their relation to the psychophysical function, see García-Pérez and Alcalá-Quintana (2007).

**Figure 3:**
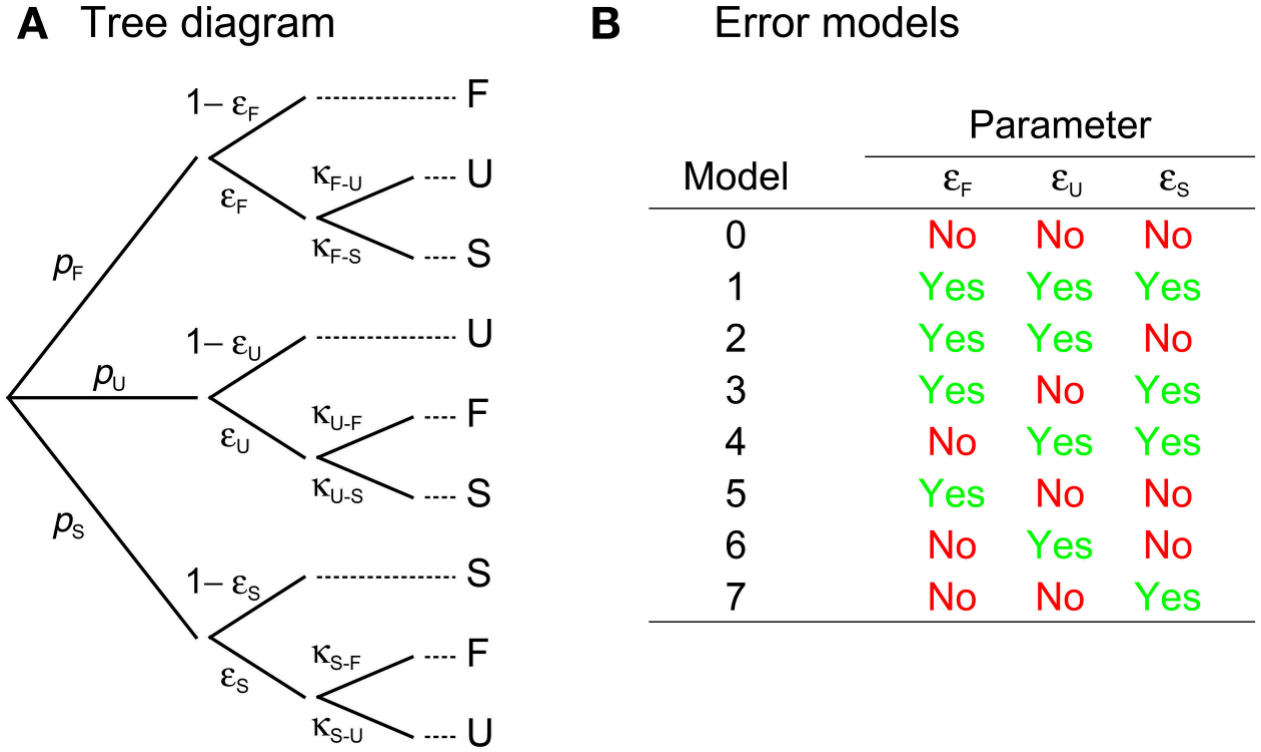
**(A)** Tree diagram describing the mapping of judgments (which occur with probabilities given by the *p*'s at the far left, given the applicable test and standard levels) onto responses (rightmost column) when response errors may occur. The subscript denoting the interval in which the test is presented has been dropped, but judgment probabilities (*p*'s) as well as error (ε's) and bias (κ's) parameters may vary across presentation orders. Recall that only one of the two κ's at each branching point is a free parameter, as they add up to unity. **(B)** Labels for error models according to whether or not they include each of the error parameters. Inclusion of an error parameter implies that its value as well as that of its associated κ must be estimated from the data for the corresponding presentation order; exclusion implies that the error parameter is assumed to be zero and, hence, that the applicable branches are removed from the tree diagram, which removes along the way the associated κ's.

Equation 3 supply the probability of judgments as a function of test level for each presentation order and they were regarded as the observable psychometric functions in the original model. But this is not necessarily true and an amendment is needed because judgments are not always reliably reported due to key-press errors or for other reasons. This amendment is analogous to the addition of lapse-rate parameters to conventional psychometric functions. Let ϵ_F,*m*_, ϵ_U,*m*_, and ϵ_S,*m*_ be the probabilities that an observer misreports F, U, and S judgments, respectively, when the test is presented in interval *m* ∈ {1, 2}. Misreporting a given judgment can take two forms. Let κ_X−Y,*m*_ be the bias toward misreporting an X judgment as a Y response when the test is presented in interval *m* so that κ_X−Z,*m*_ = 1 − κ_X−Y,*m*_ is the bias toward misreporting an X judgment as a Z response. Then, only three bias parameters are free for each presentation order, say, κ_F−U,*m*_, κ_U−F,*m*_, and κ_S−F,*m*_. Figure 3A illustrates the mapping of judgments onto responses when misreports occur. The observable psychometric functions for F, U, and S responses under each presentation order are then

(4a) $$\begin{array}{l} \Psi_{\text{F},m}\left(x\right)=\left(1-\varepsilon_{\text{F},m}\right)p_{\text{F},m}\left(x\right)+\varepsilon_{U,m}\kappa_{\text{U}-\text{F},m}p_{\text{U},m}\left(x\right)\\ +\;\varepsilon_{S,m}\kappa_{\text{S}-\text{F},m}p_{\text{S},m}\left(x\right) \end{array}$$

(4b) $$\begin{array}{l} \Psi_{\text{U},m}\left(x\right)=\varepsilon_{\text{F},m}\kappa_{\text{F}-\text{U},m}p_{\text{F},m}\left(x\right)+\left(1-\varepsilon_{U,m}\right)p_{\text{U},m}\left(x\right)\\ +\varepsilon_{S,m}\kappa_{\text{S}-\text{U},m}p_{\text{S},m}\left(x\right) \end{array}$$

(4c) $$\begin{array}{l} \Psi_{\text{S},m}\left(x\right)=\varepsilon_{\text{F},m}\kappa_{\text{F}-\text{S},m}p_{\text{F},m}\left(x\right)+\varepsilon_{U,m}\kappa_{\text{U}-\text{S},m}p_{\text{U},m}\left(x\right)\\ +\left(1-\varepsilon_{S,m}\right)p_{\text{S},m}\left(x\right), \end{array}$$

where the *p*'s come from Equations (3). Note that Equations (4) revert to Equations (3) when all ε's are zero (i.e., when judgments are never misreported, as was assumed in the original model).

Errors rarely occur in all forms under both presentation orders. Figure 3B shows that there are eight error models per presentation order according to how many and which of the ε's are included and, hence, 64 combinations across presentation orders. Model (0, 0) is the original model and involves the least number of free parameters: only two or three from the psychophysical functions (see below) plus δ_1_ and δ_2_; at the other end, model (1, 1) assumes that errors occur in all possible forms and adds six error/bias parameters per presentation order. In the analysis of empirical data, fitting model (1, 1) routinely may result in null estimates of some of the ε's, indicating that those ε's and their associated κ's should not have been included. Unnecessary parameters do not affect the quality of the fit but they have consequences for goodness-of-fit assessments. Consideration of all error models allows choosing a model without unnecessary error parameters.

The number of parameters coming from the psychophysical functions deserves commentary. When μ_t_ = μ_s_, only one set of α and β is involved (top panel in Figure 2A), potentially resulting in two free parameters. With detection data both parameters are identifiable, but α is not identifiable with suprathreshold discrimination data, as discussed above. When μ_t_ ≠ μ_s_ instead, two sets of α and β seem necessary but the use of a single standard level precludes estimating the parameters of μ_s_ because only μ_s_(*x*_s_) manifests by setting an anchor. In this case, the free parameters are μ_s_(*x*_s_), α_t_, and β_t_, although α_t_ will not be identifiable with suprathreshold discrimination data.

## Accommodating the classical 2AFC and same–different response formats

The indecision model assumes that judgments precede responses and are unaffected by how the response format asks observers to report them. Under the ternary format, judgments lead to responses as discussed in the preceding section. Under the 2AFC format, observers give F or S responses at random upon U judgments; analogously, under the same–different format, observers respond “same” upon U judgments and “different” upon F or S judgments. This allows expressing responses under these binary formats in terms of the indecision model.

**Figure 4:**
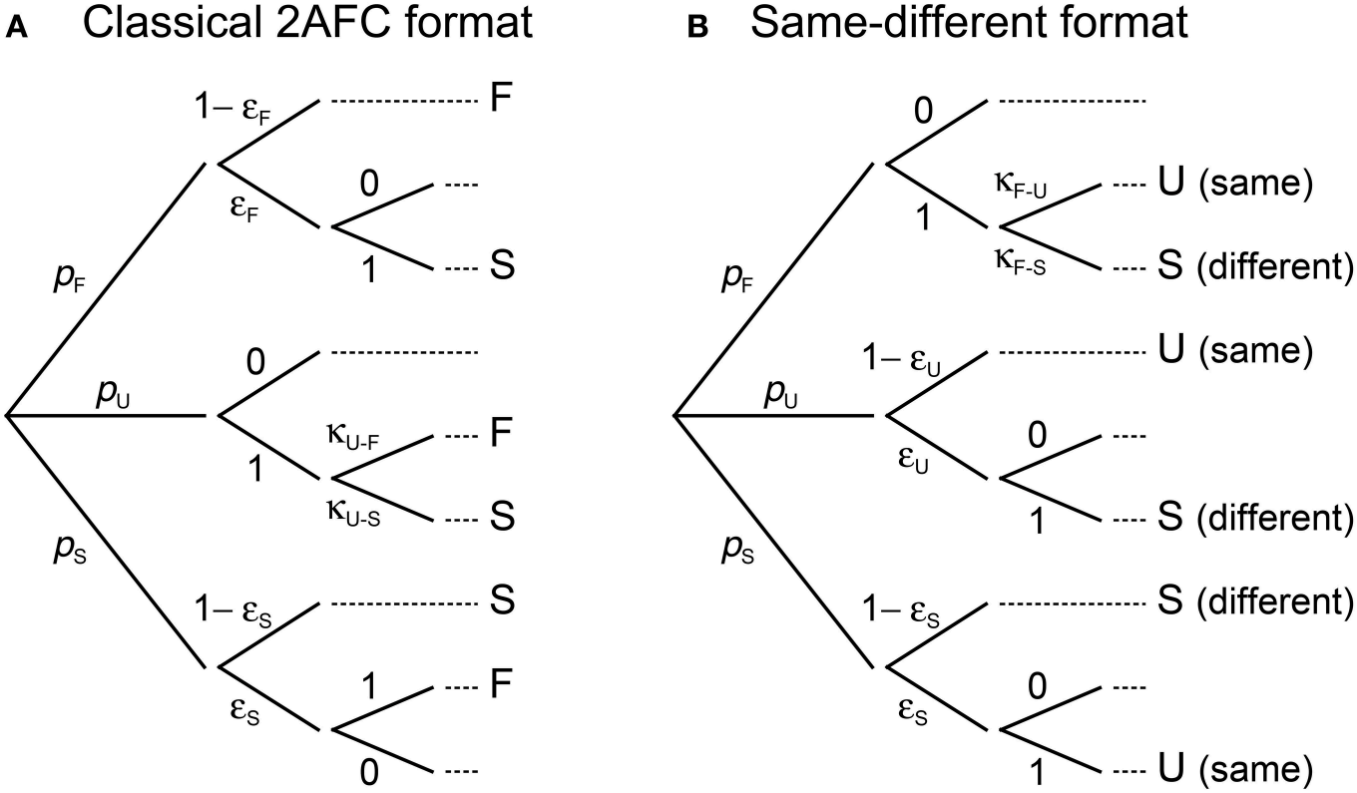
Characterization of binary response formats in terms of the ternary model. Compared to the tree diagram for the ternary format in Figure 3A, in the 2AFC format **(A)** observers behave with ε_U,*m*_ = 1, κ_F−S,*m*_ = 1, and κ_S−F,*m*_ = 1; in the same–different format **(B)** observers behave with ε_F,*m*_ = 1, κ_U−S,*m*_ = 1, and κ_S−U,*m*_ = 1. These simplifications reduce the number of free parameters in the corresponding model.

Under 2AFC responding, observers behave with ε_U,*m*_ = 1 but not necessarily with κ_U−F,*m*_ = 0.5. Also, F (S) judgments can only be misreported as S (F) responses, making κ_F−S,*m*_ = κ_S−F,*m*_ = 1. This renders the simplified diagram in Figure 4A and turns Equations (4) into

(5a) $$\begin{array}{l} \Psi_{\text{F},m}\left(x\right)=\left(1-\varepsilon_{\text{F},m}\right)p_{\text{F},m}\left(x\right)+\kappa_{\text{U}-\text{F},m}p_{\text{U},m}\left(x\right)+\varepsilon_{S,m}p_{\text{S},m}\left(x\right) \end{array}$$

(5b) $$\begin{array}{l} \Psi_{\text{U},m}\left(x\right)=0 \end{array}$$

(5c) $$\begin{array}{l} \Psi_{\text{S},m}\left(x\right)=\varepsilon_{\text{F},m}p_{\text{F},m}\left(x\right)+\kappa_{\text{U}-\text{S},m}p_{\text{U},m}\left(x\right)+\left(1-\varepsilon_{S,m}\right)p_{\text{S},m}\left(x\right), \end{array}$$

with a reduction in the number of free parameters (i.e., only two ε's and a single κ per presentation order). Thus, accommodating 2AFC responding is straightforward without changing the notation.

A change of notation seems necessary to accommodate the same–different format, though only to refer to “same” and “different” responses instead. Mapping F, U, and S judgments onto “same” or “different” responses on consideration that errors can occur turns Equations (4) into

(6a) $$\begin{array}{l} \Psi_{\text{same},m}\left(x\right)=\varepsilon_{\text{F},m}p_{\text{F},m}\left(x\right)+\left(1-\varepsilon_{\text{U},m}\right)p_{\text{U},m}\left(x\right)+\varepsilon_{S,m}p_{\text{S},m}\left(x\right) \end{array}$$

(6b) $$\begin{array}{l} \Psi_{\text{diff},m}\left(x\right)=\left(1-\varepsilon_{\text{F},m}\right)p_{\text{F},m}\left(x\right)+\varepsilon_{\text{U},m}p_{\text{U},m}\left(x\right)\\ +\;\left(1-\varepsilon_{S,m}\right)p_{\text{S},m}\left(x\right). \end{array}$$

To avoid notational changes, same–different responding can be expressed as shown in Figure 4B. Specifically, F judgments are regarded as “misreported” always (i.e., ε_F,*m*_ = 1), either as S responses (to render the aggregated “different” response category) or as “same” responses due to errors; U judgments are reported as “same” responses with probability 1 − ε_U,*m*_ or, due to errors, misreported as “different” responses with probability ε_U,*m*_; finally, S judgments are reported as “different” responses with probability 1 − ε_S,*m*_ or misreported as “same” responses with probability ε_S,*m*_. The number of free parameters is also reduced here and Equations (4) become

(7a) $$\begin{array}{l} \Psi_{\text{F},m}\left(x\right)=0 \end{array}$$

(7b) $$\begin{array}{l} \Psi_{\text{U},m}\left(x\right)=\kappa_{\text{F}-\text{U},m}p_{\text{F},m}\left(x\right)+\left(1-\varepsilon_{U,m}\right)p_{\text{U},m}\left(x\right)+\varepsilon_{S,m}p_{\text{S},m}\left(x\right) \end{array}$$

(7c) $$\begin{array}{l} \Psi_{\text{S},m}\left(x\right)=\kappa_{\text{F}-\text{S},m}p_{\text{F},m}\left(x\right)+\varepsilon_{U,m}p_{\text{U},m}\left(x\right)+\left(1-\varepsilon_{S,m}\right)p_{\text{S},m}\left(x\right). \end{array}$$

Except for notation, Equations (7) are identical to Equations (6): κ_F−U_ in Equation (7b) plays the role of ε_F_ in Equation (6a) and κ_F−S_ = 1 − κ_F−U_ in Equation (7c) plays the role of 1 − ε_F_ in Equation (6b).

Yet, a price is paid when forcing observers to misreport U judgments as F or S responses (under 2AFC responding) or to collapse F and S judgments into “different” responses (under same–different responding). Parameter estimates are likely to be less accurate because data that would have been informative separately are now mixed together. This is particularly true under 2AFC responding, where data reflect an inextricable mixture of guesses and authentic F or S responses. An unfortunate byproduct of this mix-up is that decisional and bias parameters are confounded: Observed data can be nearly identically accounted for on the assumptions that observers were never undecided (i.e., δ_1_ = δ_2_, which renders the *difference model with bias*; see Figure 3 in García-Pérez and Alcalá-Quintana, 2011a) or that they were undecided to some extent (i.e., δ_1_ ≠ δ_2_) and gave F responses with a bias captured by parameters κ_U−F,*m*_. Note that δ_1_ = δ_2_ makes *p*_U_ = 0 in the diagram of Figure 4A (see Equations 3b,e), eliminating κ_U−F,*m*_ along the way. The classical decision rule for 2AFC responding (i.e., U judgments do not occur) can thus be accommodated by the indecision model via enforcing the assumption that δ_1_ = δ_2_, which eliminates three free parameters (δ_2_, κ_U−F,1_, and κ_U−F,2_). None of this applies under same–different responding because the mere presence of “same” responses implies δ_1_ ≠ δ_2_.

It must be noted that 2AFC or same–different data should be adequately fitted by the ternary model without the modifications just discussed. The absence of U responses (in 2AFC data) or the absence of F responses (in our characterization of same–different data) should return 0's or 1's for the applicable error and bias parameters in the diagrams of Figure 4. We will show that this is the case with the examples given later in this paper, but the fact that those parameters valued at 0 or 1 are fixed and not free must be considered on assessing goodness of fit.

## Comparison of parameter estimates from ternary vs. binary data

Model presentation in the preceding sections suggests that ternary data should provide more accurate estimates of sensory and decisional parameters than binary data. The surmise gains support from the results of an analogous comparison for single-presentation tasks (where a single stimulus is presented in each trial for observers to report a judgment; see García-Pérez and Alcalá-Quintana, 2012). Evidence on the superiority of the ternary format in 2P tasks is lacking and this section reports simulation results that demonstrate it.

It is important to stress first the scope of these simulations. If data are scarce, collected at uninformative test levels, or corrupted by inappropriate experimental control, parameter estimates will be inaccurate, biased, or non-sensical. Issues such as optimal strategies to maximize the informative value of the data (e.g., adaptive data collection) or optimal sample sizes (i.e., number of test levels and number of trials per level) are not addressed in these simulations, as they do not bear on a comparison of response formats (for some results regarding those issues, see, e.g., Dai, 1995; Lam et al., 1996, 1999; García-Pérez and Alcalá-Quintana, 2005; Chaudhuri and Merfeld, 2013; García-Pérez, 2014b; Karmali et al., 2016). The goal of these simulations is instead to assess parameter recovery when sufficient data are collected at informative test levels. The simulations assess the ability to estimate relevant parameters when α_t_ is not identifiable and, more generally, the relative precision of parameters estimated from ternary data vs. 2AFC or same–different data.

To make results comparable across conditions, the same true parameters (which varied across 2,000 replicates) were used in seven scenarios resulting from a combination of tasks (detection or discrimination) and response formats (ternary, 2AFC, or same–different): ternary detection, 2AFC detection, ternary discrimination with μ_s_ = μ_t_, 2AFC discrimination with μ_s_ = μ_t_, ternary discrimination with μ_s_ ≠ μ_t_, 2AFC discrimination with μ_s_ ≠ μ_t_, and same–different discrimination with μ_s_ = μ_t_. The context of reference is visual contrast perception but the results do not depend on context. True parameters were drawn from uniform distributions on [−3, −2] for α_t_, on [0.05, 0.10] for β_t_, on [−4, −2] for δ_1_, and on [2, 4] for δ_2_. In scenarios involving discrimination with μ_s_ ≠ μ_t_, the anchor μ_s_(*x*_s_) was drawn from a uniform distribution on [μ_t_(*x*_s_) − 1, μ_t_(*x*_s_) + 1]. Simulations were run under error model (1, 1) with error and bias parameters drawn from uniform distributions on [0, 0.02] and [0, 1], respectively, and also under error model (0, 0) with all error parameters set to 0. In scenarios involving binary formats, the applicable error and bias parameters were set to the fixed values that hold in each case (Figure 4).

In all scenarios, responses were simulated to 40 trials at each of the same 11 test levels for each presentation order. For detection, the central test level was the true α_t_ in the current replicate rounded to the nearest multiple of 0.1 whereas, for discrimination, the central test level was the standard level *x*_s_ = −1 (a suprathreshold level given the ranges of α_t_ and β_t_); in either case, the remaining levels moved out in steps of 0.1 units in each direction. Note that the constant spacing of test levels is not adjusted to the steepness of μ_t_ (i.e., the true value of β_t_) in each replicate.

Maximum-likelihood parameter estimates were obtained for each replicate with the method described in the next section, using multiple starting points to minimize the chances of missing the global optimum (further details are given in the Supplementary Material). Detection thresholds or PSEs (as applicable) were subsequently obtained from parameter estimates using the expressions given earlier and compared with the values identically obtained from true parameters.

**Figure 5:**
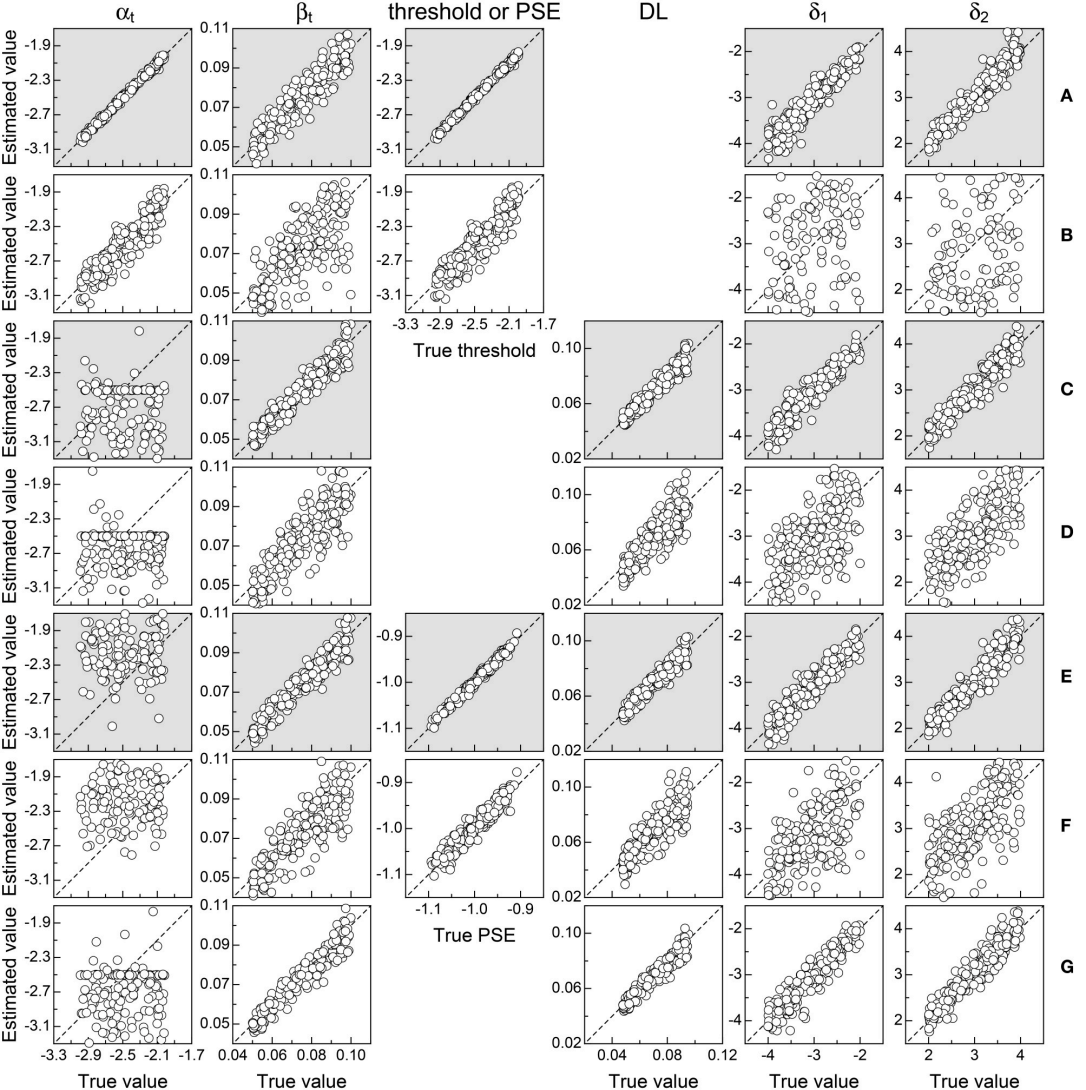
Scatter plots of estimates against true parameters in each scenario (rows) for each relevant model parameter (columns; see labels at the top), including performance measures (detection threshold, PSE, and DL; center columns). The dashed diagonal is the identity line; grayed panels denote conditions involving the ternary format. To avoid clutter, symbols are plotted for a random subset of 200 replicates. **(A)** Ternary detection. **(B)** 2AFC detection. **(C)** Ternary discrimination with μ_s_ = μ_t_. **(D)** 2AFC discrimination with μ_s_ = μ_t_. **(E)** Ternary discrimination with μ_s_ ≠ μ_t_. **(F)** 2AFC discrimination with μ_s_ ≠ μ_t_. **(G)** Same–different discrimination with μ_s_ = μ_t_.

Figure 5 shows the results in the form of scatter plots of parameter estimates against true values in each scenario (rows) for data without response errors. Consider Figure 5A first, for ternary detection data. The tight packing of symbols along the identity line reveals that all parameters were reasonably well-estimated, also resulting in accurate estimates of the detection threshold (center panel in the row). In comparison, 2AFC data collected under identical conditions (Figure 5B) rendered less accurate estimates, attesting to the inferiority of a response format in which informative F and S responses are mixed up with uninformative guesses. Note that δ_1_ and δ_2_ are also very poorly estimated in this case, owing to the confound with the bias for misreporting U judgments: Multiple sets of estimates of δ_1_, δ_2_, κ_U−F,1_, and κ_U−F,2_ (with δ_1_ = δ_2_ or δ_1_ ≠ δ_2_) produce curves that fit the data equally well. A detailed illustration of this characteristic is given below.

A comparison of the outcomes for ternary vs. 2AFC data in suprathreshold discrimination with μ_s_ = μ_t_ (Figures 5C,D) or with μ_s_ ≠ μ_t_ (Figures 5E,F) offers the same picture: All else equal, estimates from 2AFC data are less accurate than estimates from ternary data. Note that in the four cases under discussion, estimates of α_t_ are very poor compared to those obtained from detection tasks (Figures 5A,B). This evidences the unidentifiability of α_t_, which does not play any role in suprathreshold discrimination and, hence, cannot be estimated. Interestingly, the unidentifiability of α_t_ does not affect estimation accuracy for the remaining parameters, which varies only with the response format used to collect data. Also, a comparison of the panel for β_t_ in Figure 5A (ternary detection) with those in Figures 5C,D (ternary discrimination) reveals that β_t_ is more accurately estimated with discrimination tasks. This is understandable because detection tasks probe the non-linear range of μ_t_, which is less informative of β_t_ than the linear range probed in suprathreshold discrimination tasks.

Finally, results for same–different data (Figure 5G) fall between those for ternary (Figure 5C) and 2AFC (Figure 5D) data in analogous conditions. This is because aggregating F and S responses into the “different” category is less detrimental than corrupting F and S responses by distributing U judgments at random between them. Poor estimation of α_t_ here is also due to the fact that it does not play any role in suprathreshold discrimination. Finally, note that β_t_ is estimated here with about the same precision provided by ternary data, and the same holds for estimates of δ_1_ and δ_2_ because the same–different format does not confound decisional and bias parameters.

Results for data simulated under error model (1, 1) displayed the same trends, although the presence of response errors deteriorated estimation accuracy proportionately in all scenarios. These results are presented in the Supplementary Material.

In sum, model parameters can be estimated more accurately from ternary data than from 2AFC or same–different data. Because the numbers of stimulus levels and trials per level were identical with all formats, empirical cost and burden do not vary with response format and, hence, these results identify the ternary format as the most efficient strategy to collect psychophysical data.

## Fitting the ternary indecision model

Harvesting the benefits of the ternary response format requires custom software to estimate model parameters. The Supplementary Material includes matlab (http://www.mathworks.com) and R (http://cran.r-project.org) routines that accomplish this goal, usage documentation, and scripts to run the examples in the next section. It should be noted that this software fits the indecision model (extended to incorporate the error model of choice) with the constraints that hold for detection or discrimination data, with the constraints that hold according to the response format, and under the user-selected assumption about (in)equality of μ_t_ and μ_s_. The software also fits 2AFC data under the alternative assumptions that δ_1_ = δ_2_ or δ_1_ ≠ δ_2_. This flexibility permits direct tests of certain hypotheses, although the next section will demonstrate that 2AFC data are unsuitable for these purposes. For an overview of these features, we will briefly describe the matlab script in Exhibit 1 and its outcomes, which also serves to introduce the examples to come in the next section.

**Exhibit 1:**
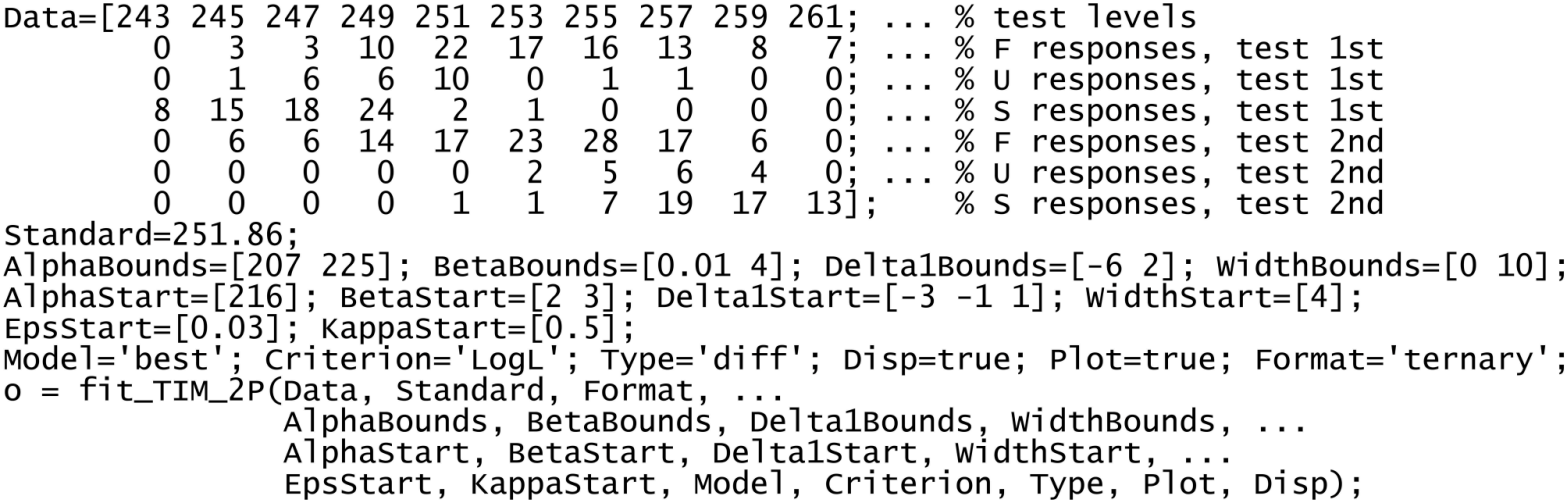
**matlab** script to fit the indecision model.

Data come from one of the observers in one of the conditions in García-Pérez and Peli (2015), which involved suprathreshold discrimination with standard and test stimuli for which μ_t_ ≠ μ_s_. The data (first assignment in the script) are arranged in an array with as many columns as levels had the test stimulus and with seven rows containing the set of test levels that were used (first row) and the counts of F, U, and S responses at each level when the test was presented first (rows 2–4) and second (rows 5–7). The next line defines the standard level, implicitly indicating that the data come from a discrimination task.

**Figure 6:**
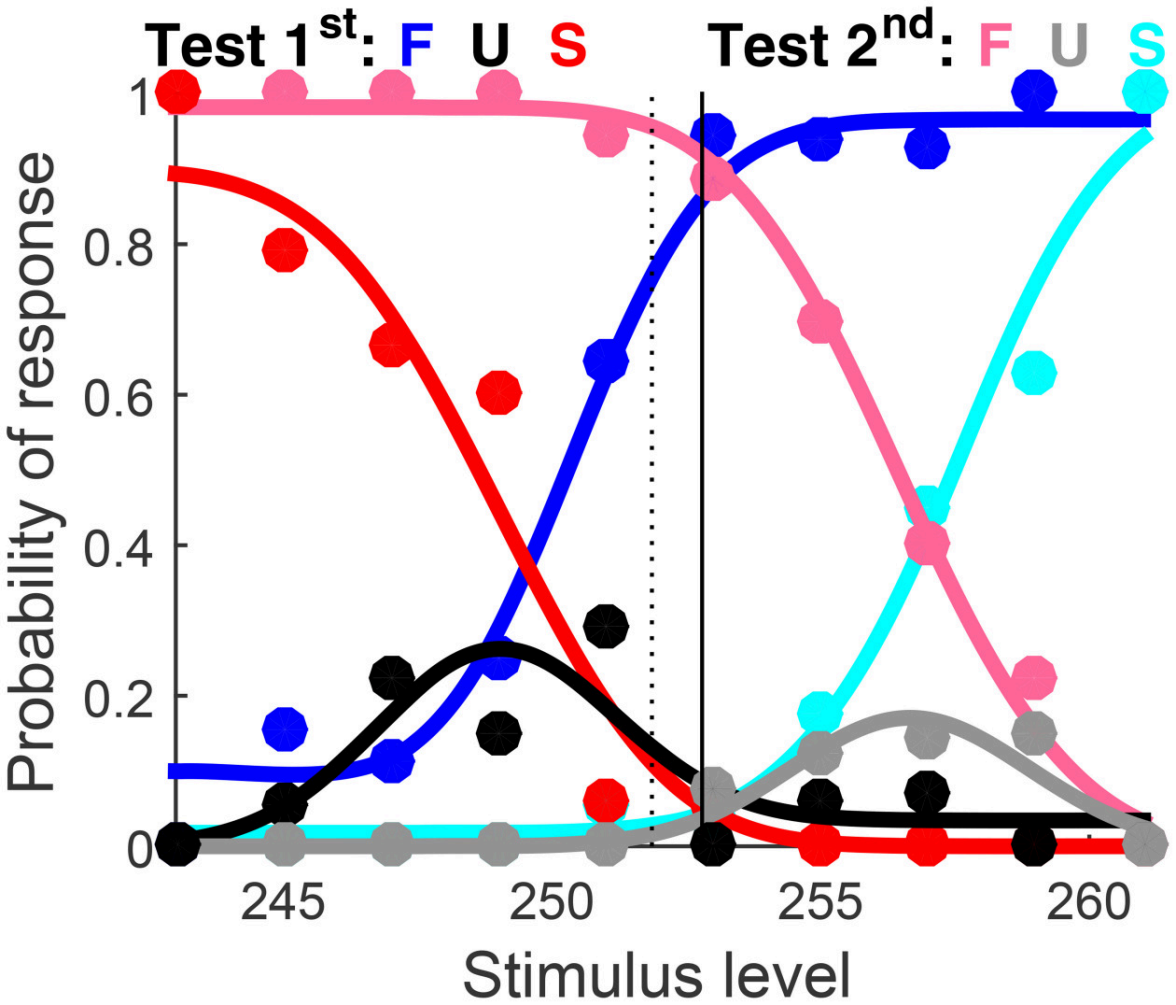
Graphical output. Color codes for data points and functions are indicated at the top. The horizontal axis spans the range of test levels. If data come from a discrimination task, as in this case, a vertical dashed line indicates the level of the standard stimulus and a solid vertical line indicates the location of the PSE.

**Figure 7:**
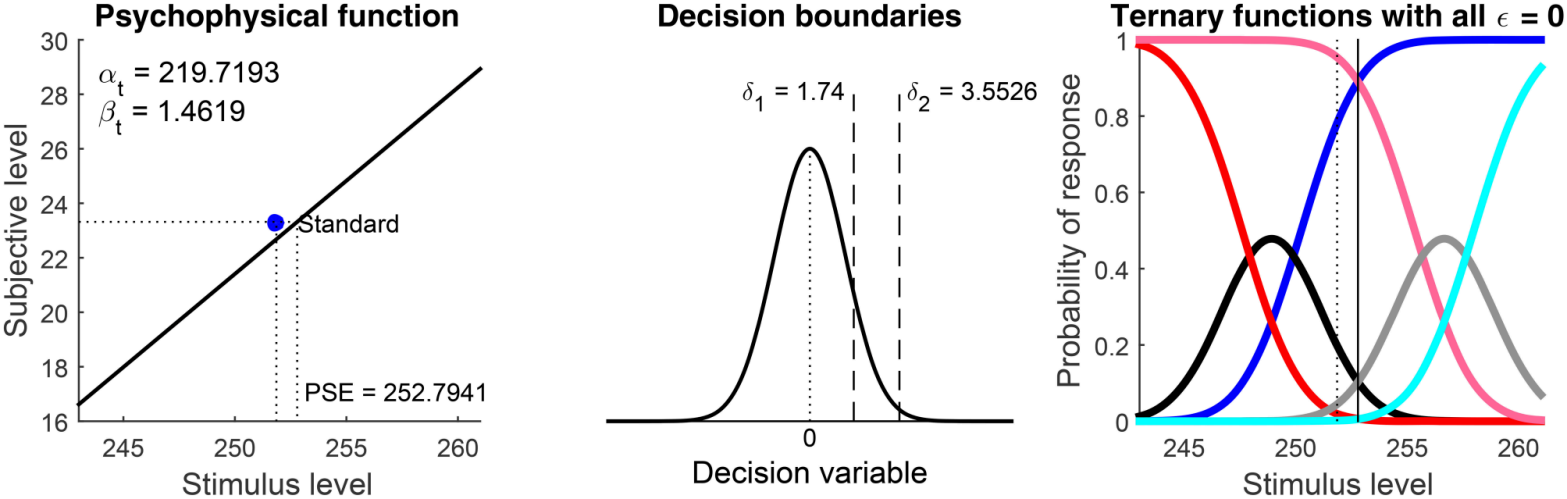
Additional graphical output. The left panel plots the estimated psychophysical function μ_t_ for the test, with parameters given in the panel. The horizontal axis spans the range of test levels. If data come from a discrimination task, as in this case, a blue dot at coordinates (*x*_s_, μ_s_(*x*_s_)) indicates the estimated subjective level of the standard. The blue dot will generally not lie on μ_t_ if the latter was assumed to differ from μ_s_. The central panel depicts the estimated boundaries in decision space, plotting also for reference the distribution of the decision variable at the test level *x* such that μ_t_(*x*) = μ_s_(*x*_s_) (i.e., a Gaussian with mean 0 and variance 2). The right panel depicts the (latent) psychometric functions that would have been observed in the absence of response errors, that is, the probabilities of judgments according to Equations (3).

**Figure 8:**
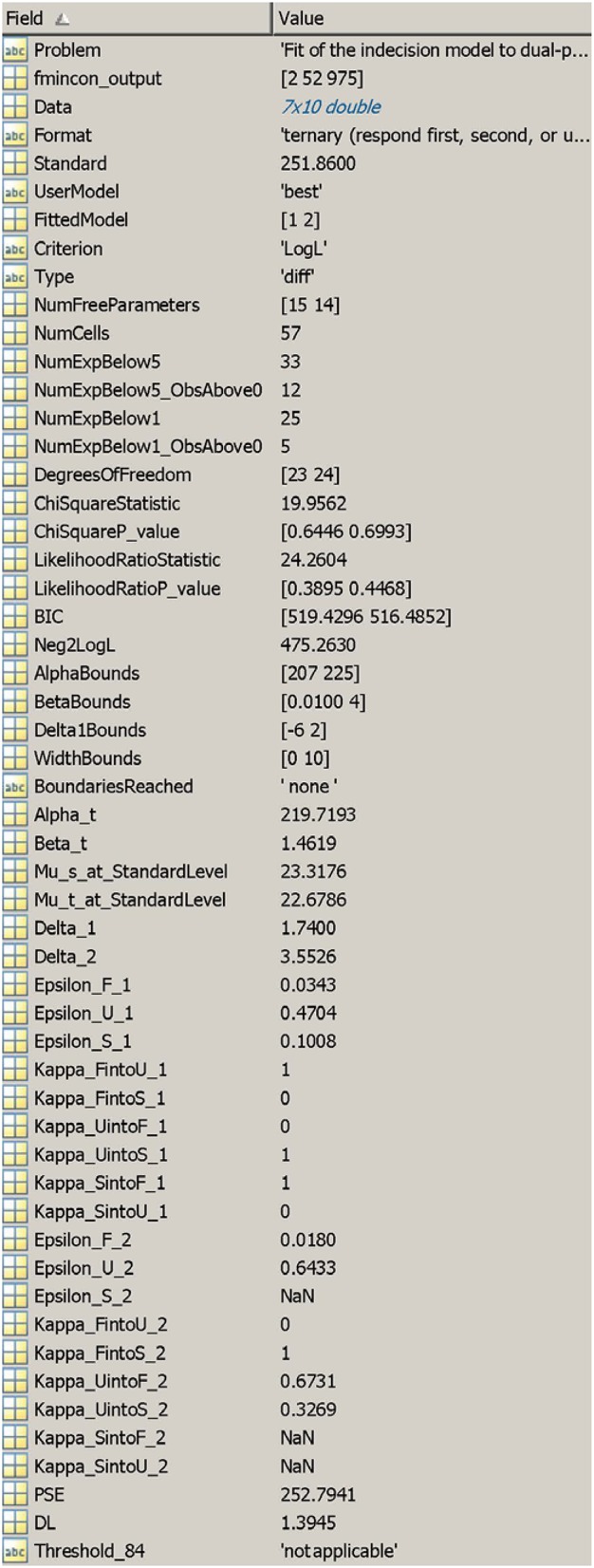
Output structure from the **matlab** function. The output list from the R function is analogous.

The next line bounds the search space for parameters α_t_, β_t_, and δ_1_ and also for the width δ_2_ – δ_1_. Bounds for the ε's and κ's are well-defined as 0 and 1 without user intervention. Bounds are required by the optimization algorithm and issues involving their choice are addressed in the usage documentation. Maximum-likelihood estimates are sought with the matlab built-in function fmincon or the R built-in function optim (for further details, see footnote 2 in Alcalá-Quintana and García-Pérez, 2013). These functions also require starting values for each parameter and they are not guaranteed to return the global optimum; then, the script defines several starting values for some parameters, which are factorially combined to obtain a solution for each multidimensional starting point thus defined so as to return the optimal solution across the board. Starting values are defined in the next two lines in the script: a single value for α_t_, two for β_t_, three for δ_1_, and one for the width δ_2_ – δ_1_, for the ε's, and for the κ's. The next line sets additional arguments so that the routine returns the best-fitting error model according to the log-likelihood of the data, fits the data under the assumption that psychophysical functions differ for standard and test, displays progress information during its operation, and plots results upon completion; the last assignment states that data were collected with the ternary response format, which anticipates that the same routine fits data collected with the 2AFC or the same–different formats. (For a thorough description of each of these arguments and their functionality, see the usage documentation in the Supplementary Material.) The function is called with these arguments in the last line. The output returned in o is a structure (in matlab) or a list (in R) including parameter estimates and complete information about the results. This script produces the plots in Figures 6, 7 and the structure in Figure 8.

The output (Figure 8) includes a label for the problem (field 1) and diagnostic information from fmincon (output flag, number of iterations, and number of function evaluations, in a row vector; field 2),^1^ the data, the response format, and the standard level (fields 3–5), the user-selected error model and the model for which parameters are returned (fields 6 and 7), the criterion selected to search for the best-fitting model, if applicable (field 8), the type of fit regarding psychophysical functions for test and standard (field 9), the number of free parameters in the fitted model (field 10), the total number of cells for goodness-of-fit tests, the number of cells in which expected frequencies were smaller than 5 and the number of those cells in which observed frequencies were non-null (fields 11–13), the number of cells in which expected frequencies were smaller than 1 and the number of those cells in which observed frequencies were non-null (fields 14 and 15), the degrees of freedom, values, and *p*-values of Pearson's chi-square (*X*^2^) and the likelihood-ratio (*G*^2^) goodness-of-fit statistics (fields 16–20), the Bayesian information criterion (BIC) of the fitted model (field 21), the −2Log*L* of the data under the fitted model (field 22), the user-defined content of AlphaBounds, BetaBounds, Delta1Bounds, and WidthBounds (fields 23–26), a statement indicating which boundaries were reached, if any (field 27), estimates for α_t_ and β_t_ (fields 28 and 29), the estimated anchor μ_s_(*x*_s_) and the ordinate of μ_t_ at *x* = *x*_s_ (fields 30 and 31), estimates of δ_1_ and δ_2_ (fields 32 and 33), estimates of the ε and κ parameters when the test was presented first (fields 34–42) and second (fields 43–51), and performance measures (PSE, DL, and detection threshold) as applicable (fields 52–54). Comments regarding these fields are given in the usage documentation, including specificities that apply to 2AFC or same–different data.

## Annotated examples using published data

The following examples illustrate and discuss the fitting of the indecision model to data from detection tasks, from discrimination tasks in which the same or different psychophysical functions hold for standard and test, with diverse ranges and scales for test levels, and for data collected with the ternary, 2AFC, or same–different formats. These examples illustrate and discuss the various theoretical options described above to fit ternary, 2AFC, and same–different data, also proving the impossibility to test certain types of hypotheses with 2AFC data. Parameter estimates were obtained with the routine described in the preceding section. All examples use published data that had been analyzed differently in the original sources and each example starts describing relevant aspects of data collection and analysis in each study.

As seen in the accompanying scripts, BetaBounds, Delta1Bounds, WidthBounds, BetaStart, Delta1Start, WidthStart, EpsStart, and KappaStart were set as in Exhibit 1 and they will not be mentioned again (except for WidthBounds and WidthStart in example 4bis to fit 2AFC data enforcing δ_1_ = δ_2_). Criteria used to set these arguments are discussed in the usage documentation. AlphaBounds was set differently in each example using a simple criterion that will be discussed here. AlphaStart was always set to a scalar at the midpoint of AlphaBounds. Standard, Format, and Type were set as needed in each example, as they embody theoretical and empirical options to fit the model. Finally, because model selection is not an issue here, Model = 1 was used. All the examples show output of the matlab routine; comments regarding the output produced by the R version are given in the usage documentation.

### Example 1. visual detection of contrast; ternary responses

Data for this example come from a study on contrast detection of Gabor patches (García-Pérez et al., 2011). In different conditions, the target was or was not flanked by suprathreshold patches. Ternary data were collected with a temporal 2P task but U responses were immediately treated as suggested by Fechner (1860/1966), namely, counting them as half correct and half incorrect to render binary data. In some analyses, logistic psychometric functions were fitted to data aggregated across presentation orders (see Figure 4A in García-Pérez et al., 2011). This example fits instead the indecision model to the original ternary data from observers M1, M2, and M3 in the non-flanked condition. Test levels (log contrast) varied across observers due to the adaptive collection of data, but they ranged from −2.45 to −1.55 across the board. The overall number of trials ranged from 884 to 915 across observers and were distributed unevenly across test levels and presentation orders due to the adaptive collection of data.

The script set Standard = −Inf to indicate detection data (see the usage documentation). As for AlphaBounds, the general rule for detection data was used, which consists of setting the lower bound at 3*x*_1_ − 2*x*_*N*_ and the upper bound at *x*_*N*_, using for each observer the lowest (*x*_1_) and highest (*x*_*N*_) test level in the first row of Data.

**Figure 9:**
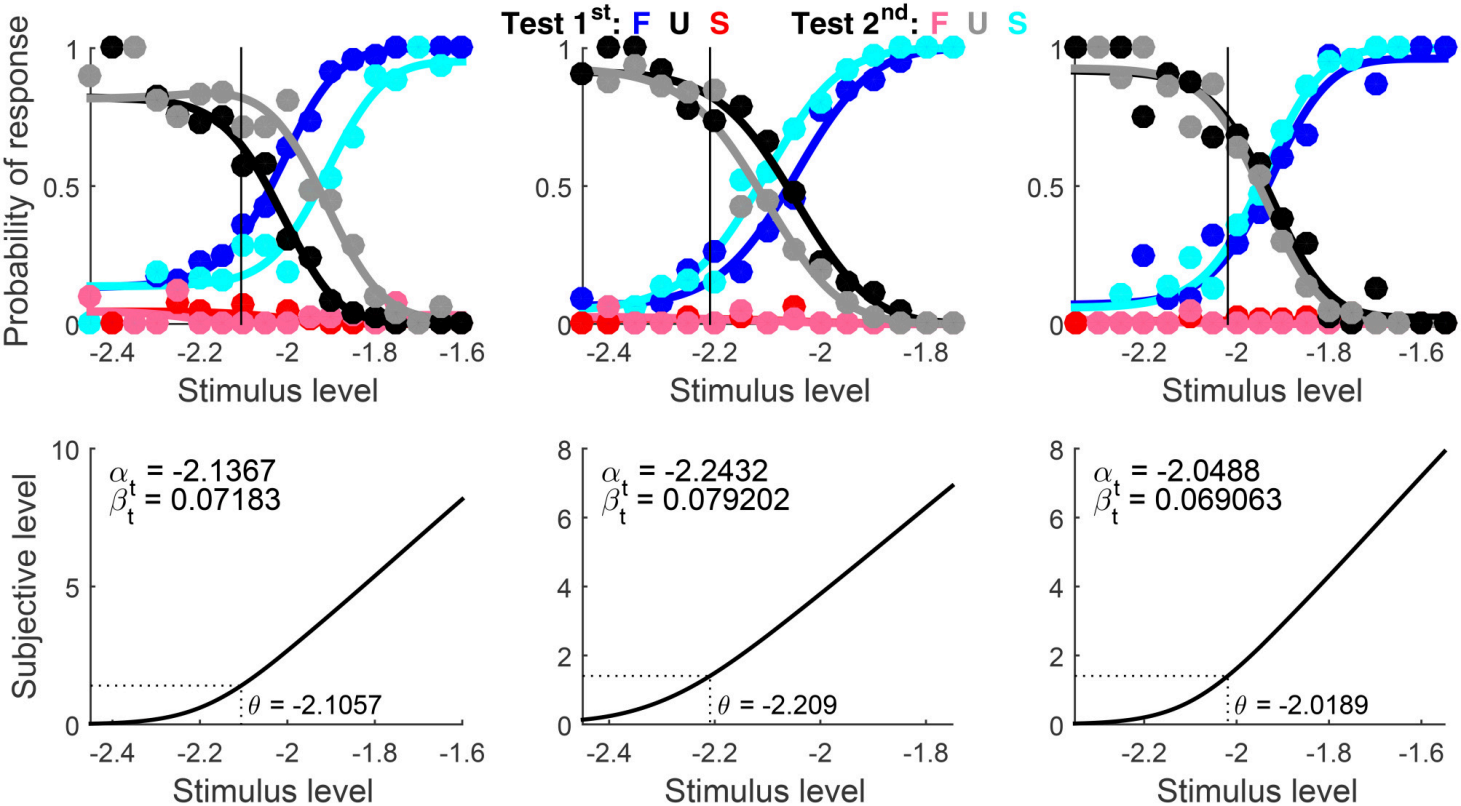
Rearranged graphical output for example 1, involving detection data collected from three observers (columns) with a ternary response format. Goodness-of-fit statistics did not reject the fitted model for any observer.

Graphical results are shown in Figure 9 in a compact form different from that which the function produced (Figures 6, 7 above). The detection threshold θ is shown in the bottom panels and marked by a vertical line in the upper panels. The upper panels thus show where θ lies relative to the rising portions of the psychometric functions for correct responses (blue and cyan curves) and the lower panels show that α_t_ is always slightly below θ. Indeed, $\theta =\mu_{\text{t}}^{-1}\left(z_{0.84}\sqrt{2}\right)=\mu_{\text{t}}^{-1}\left(1.406\right)$ whereas $\alpha_{\text{t}}=\mu_{\text{t}}^{-1}\left(\log\left(3\right)\right)=\mu_{\text{t}}^{-1}\left(1.099\right)$ (see Figure 1B). Then, a mere look at detection data informs of suitable bounds for α_t_. Recall also that α_t_ is identifiable in detection tasks, which probe the non-linear range of μ_t_ (as is evident in the bottom panels of Figure 9).

Note that incorrect responses (red and pink data points and curves in Figure 9) are rarely given under the ternary format, the natural consequence of allowing observers to report indecision instead of forcing them to guess (which makes them haphazardly and uninformatively correct or incorrect). Understandably, U responses (black and gray data points and curves) prevail at low test levels and they progressively give way to correct responses (blue and cyan data points and curves) as test level increases. Also, psychometric functions for test-first presentations (dark data points and curves) and test-second presentations (pale data points and curves) are displaced from one another in one direction for the first observer (left column), displaced in the opposite direction for the second observer (center column), and superimposed for the third observer (right column). These are the signatures of decisional bias (or lack thereof) illustrated in Figure 2 above.

### Example 2. visual discrimination of contrast; ternary responses

Suprathreshold discrimination data for this example come from the same study, observers, and condition, but for the highest standard used with each observer (namely, −0.65, −0.75, and −0.60; see Table 1 in García-Pérez et al., 2011). Data had been originally analyzed as described in the preceding example. Test levels varied across observers for the same reason, but they ranged from −1.275 to −0.15 across the board. The overall number of trials ranged between 324 and 341 across observers, for reasons described in the preceding example.

The script set Standard to the appropriate level for each observer. Because standard and test were identical except for contrast, the script set Type = ‘same’ (see the usage documentation). The true α_t_ is well below the lowest test level used in a suprathreshold discrimination task, but this parameter is unidentifiable (Figure 1C). Although AlphaBounds could be set as in example 1, a more appropriate rule for suprathreshold discrimination data sets the lower bound still at 3*x*_1_ − 2*x*_*N*_ but the upper bound at 2*x*_1_ − *x*_*N*_ instead (i.e., as far below *x*_1_ as *x*_*N*_ is above *x*_1_). Note that AlphaBounds in Exhibit 1 was set with this rule. Use of this rule ensures that μ_t_ is linear over the range of test levels, thus preventing the optimization algorithm from getting trapped around a potential local optimum at an inadequately large α_t_.

**Figure 10:**
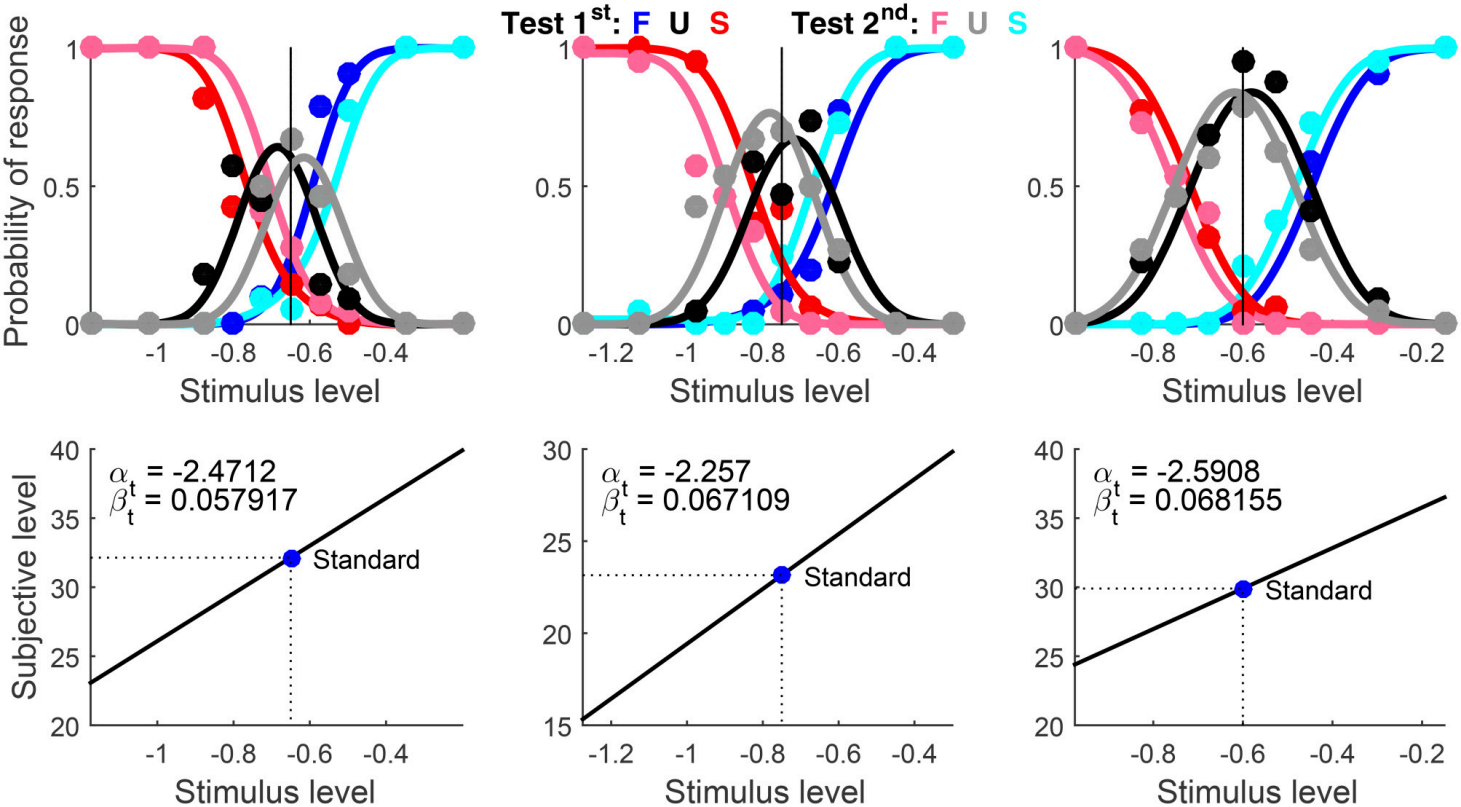
Rearranged graphical output for example 2, involving discrimination data collected from three observers (columns) with a ternary response format. Standard and test stimuli were identical except along the dimension of comparison and, hence, a common psychophysical function holds. Goodness-of-fit statistics did not reject the fitted model for any observer.

Results are shown in Figure 10 in compact form. The blue circle depicting the standard in the bottom panels lies on μ_t_ and the PSE is not reported because Type = ‘same’ implies *x*_PSE_ = *x*_s_. Recall that α_t_ is unidentifiable and does not contribute to the fit. Then, arbitrary estimates of α_t_ (bottom panels in Figure 10) do not match the dependable estimates obtained from detection data for the same observers (Figure 9). There are also discrepancies with the estimates of β_t_ from detection data for the same observers (Figure 9), surely reflecting the differences that the simulation results in Figure 5 revealed for estimates of β_t_ from detection tasks (which are not very informative about β_t_) and from informative discrimination tasks.

Decisional bias is also seen to vary across observers in these results, both in direction and in magnitude. In addition, the different height and breadth of the patterns of U responses (black and gray data points and curves) reflects the extent to which observers were undecided at test levels in the vicinity of the standard, an extent captured by the distance between estimated δ_1_ and δ_2_. (These distances cannot be appreciated in the simplified plots of Figure 10 but they are displayed in the original form of the plots created by the software, as seen in Figure 7; in general, the larger the distance between δ_1_ and δ_2_, the taller and broader the psychometric function for U responses.)

### Example 3. visual discrimination of line length; ternary responses

Data for this example come from a study on the perceived length of vertical and horizontal lines (García-Pérez and Alcalá-Quintana, 2011b). Data from two observers in the spatial discrimination task were presented in Table 2 of that paper, separated by the location in which the vertical (test) line was presented. Overall, 100 trials were administered with each presentation order at each test level. The original analyses kept presentation order separate but U responses were also treated with Fechner's method to render binary data before fitting logistic psychometric functions that satisfy theoretical constraints on slopes and locations (see Figure 4 in García-Pérez and Alcalá-Quintana, 2011b). A re-analysis (see Figure 5 in García-Pérez and Alcalá-Quintana, 2013) fitted the indecision model to the original ternary data using what we call error model (0, 0) here.

**Figure 11:**
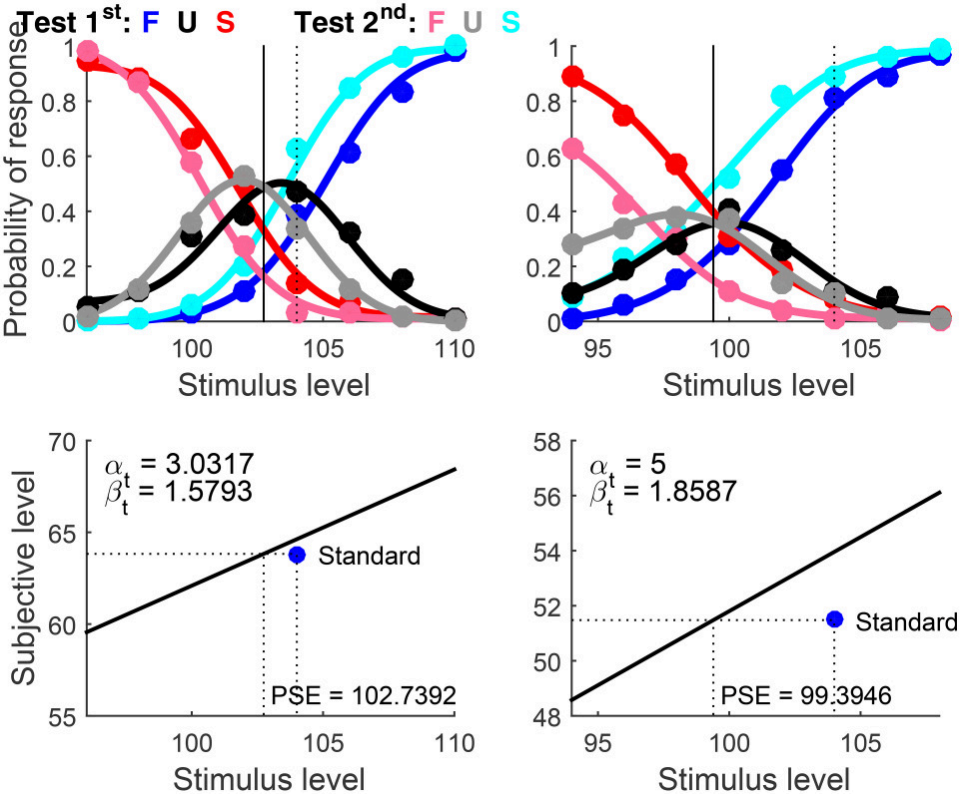
Rearranged graphical output for example 3, involving discrimination data collected from two observers (columns) with a ternary response format. Besides the dimension of comparison (i.e., length), standard and test stimuli differed in orientation and, hence, data were fitted under the assumption that different psychophysical functions hold for test and standard. Goodness-of-fit statistics did not reject the fitted model for any observer.

The script that fits error model (1, 1) instead set Standard = 104 (i.e., the length of the standard horizontal line, in pixels) and Type = ‘diff’ because the psychophysical function relating perceived length to physical length varies with line orientation (Armstrong and Marks, 1997). The detection threshold for length lies at the spatial resolution limit of the visual system and, thus, in the current units (pixels) the true α_t_ is surely below unity, although its value is impossible to estimate from suprathreshold discrimination data. The script set AlphaBounds = [−5 5], although the general rule for discrimination data discussed in example 2 could also have been used. Results are shown in Figure 11. Because α_t_ is unidentifiable, the fact that its estimate hits the upper bound in the right column of Figure 11 should not be regarded as a problem that calls for a rerun with broader bounds.

Interpretation of the results in terms of decisional bias and extent of indecision is as in the preceding example. On another front, it is clear beforehand that these data must be accounted for with μ_t_ ≠ μ_s_, as this is an established fact for perceived line length. Yet, it may not be immediately obvious in other cases whether μ_t_ ≠ μ_s_ holds. A comparison of the results returned by otherwise identical scripts that set Type = ‘diff’ and Type = ‘same’ should be informative on this issue. Re-running the script for this example with the latter option understandably results in an awful fit, whether judged by eye or via goodness-of-fit statistics. It should be stressed that the ternary format must be used for testing the alternative hypotheses that μ_t_ = μ_s_ or μ_t_ ≠ μ_s_ when the perceptual relevance of the extra dimension on which test and standard differ in a discrimination task is unclear. The reason is that U responses establish that δ_1_ ≠ δ_2_ (whose values must be estimated still), thus eliminating the confound present in 2AFC discrimination data. We will come back to this issue in the discussion of example 6 below.

### Example 4. visual detection of contrast; 2AFC responses (U not allowed)

Data for this example come from a study on contrast detection with the 2AFC format in a temporal 2P task (García-Pérez, 2000). The target was a Gabor patch and 350 trials were administered at each test level. Presentation order was randomized and, hence, the numbers of trials with each presentation order were not identical at each test level. The original analysis fitted a Weibull psychometric function to data aggregated across presentation orders (see Figure 8 in García-Pérez, 2000). To fit the indecision model here, the script set Standard = −Inf (to indicate detection data) and Format = ‘2AFC’ (see the usage documentation). AlphaBounds was set for each observer with the general rule discussed in example 1 for detection data.

**Figure 12:**
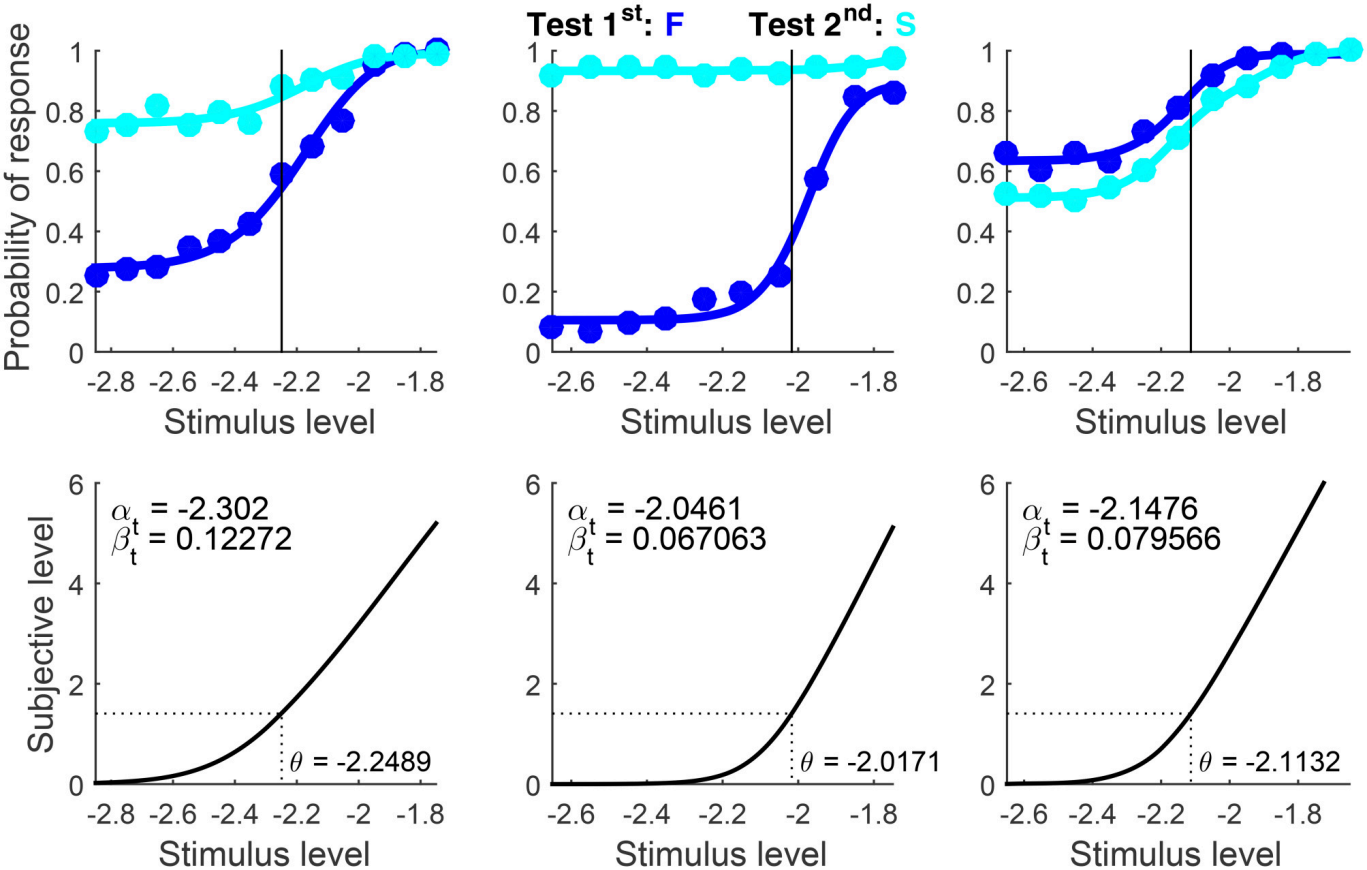
Rearranged graphical output for example 4, involving detection data collected from three observers (columns) with the classical binary response format in which observers are asked to guess when uncertain. Goodness-of-fit statistics rejected the fitted model for the first observer, despite the close correspondence between the path of the data and the fitted curves.

Results are shown in Figure 12. Only data and psychometric functions for correct responses (i.e., F responses when the test was first and S responses when it was second) are plotted, as U data and functions are trivially zero under 2AFC responding whereas data and functions for incorrect responses are redundant.

We mentioned above that the intact indecision model should fit 2AFC data identically. Readers can confirm this by re-running the script after setting Format = ‘ternary’. The resultant plots differ by showing data and psychometric functions for all three response categories, but fitted curves for F (or S) responses when the test was first (or second) are identical to those in Figure 12. Yet, since 2AFC data confound decisional and bias parameters, parameter estimates from 2AFC and ternary fits differed slightly. Differences were large only for the second observer due to uninformative data from test-second presentations (cyan curve and data points in the center column of Figure 12), which describe an essentially flat pattern compatible with multiple parametric solutions. Recall also that the reported counts of free parameters and degrees of freedom and the reported *p*-values are incorrect when the ternary model is fitted to binary data such as these.

**Figure 13:**
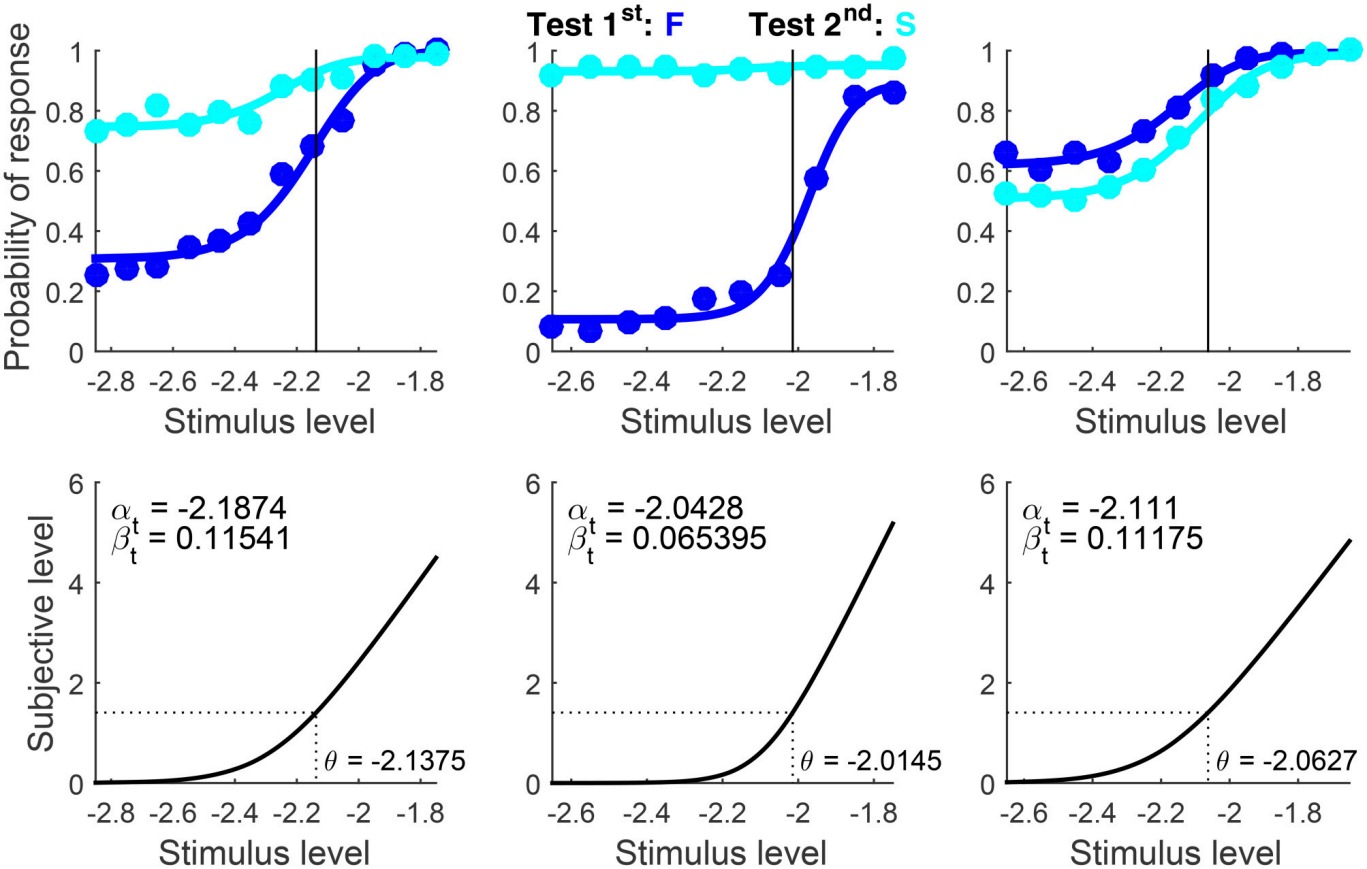
Rearranged graphical output for example 4bis, which fits the data in Figure 12 under the assumption that δ_1_ = δ_2_. As in Figure 12, goodness-of-fit statistics rejected the fitted model for the first observer despite the close agreement between data and fitted curves.

When data are collected with the 2AFC response format, information is lacking as to whether observers were ever undecided. Results in Figure 12 account for the data on the assumption that they were, via suitable estimates of δ_1_, δ_2_, κ_U−F,1_ and κ_U−F,2_. Accounting for 2AFC data in this way often results in different estimates of δ_1_ and δ_2_. Due to the confound of decisional and bias parameters, 2AFC data might also be accounted for on the assumption that δ_1_ = δ_2_ instead (i.e., observers were never undecided), which eliminates three parameters (δ_2_, κ_U−F,1_, and κ_U−F,2_ are not free parameters in this case, as discussed earlier). This assumption is enforced by setting WidthBounds = [0 0] and, naturally, WidthStart = 0 (see the usage documentation). The corresponding script is included as example 4bis and produces the output shown in Figure 13.

Compared to Figure 12, curves follow the path of the data from each observer nearly identically but with different estimates of α_t_ and β_t_ (besides δ_1_ and δ_2_) and, hence, yielding different estimates of θ. Output field 27 (BoundariesReached; see Figure 8) reported that the upper bound for width was hit for all observers, indicating that the optimization algorithm expected to find a better fit if δ_2_ > δ_1_ were allowed. Ignoring this indication, one could use the BIC to identify whether the fit with δ_1_ = δ_2_ or that with δ_1_ ≠ δ_2_ accounts better for the data from each observer, but this approach has inescapable problems and is inconclusive (see García-Pérez, 2017). Use of the ternary format in place of the 2AFC format is surely the way around this ambiguity, as U responses directly inform about indecision and its prevalence as a function of test level (as shown in example 1).

### Example 5. visual discrimination of contrast; 2AFC responses (U not allowed)

Data for this example come from a study about order effects in contrast discrimination (Alcalá-Quintana and García-Pérez, 2011). Test and standard stimuli differed only in contrast, with *x*_s_ = −1 for all observers (a level that was above the detection threshold). One of the conditions used the 2AFC format. A total of 240 trials were deployed per presentation order using adaptive methods, which unevenly distributed trials across test levels and called for different test levels with each presentation order. Across observers, test levels ranged between −1.35 and −0.65. The original analyses fitted logistic functions separately to data from each presentation order and to data aggregated across presentation orders (see Figure 6 in Alcalá-Quintana and García-Pérez, 2011). To fit the indecision model to these data, the script set Standard = −1 (i.e., the contrast of the standard stimulus), Format = ‘2AFC’, and Type = ‘same’. AlphaBounds was set with the general rule for suprathreshold discrimination data described in example 2.

**Figure 14:**
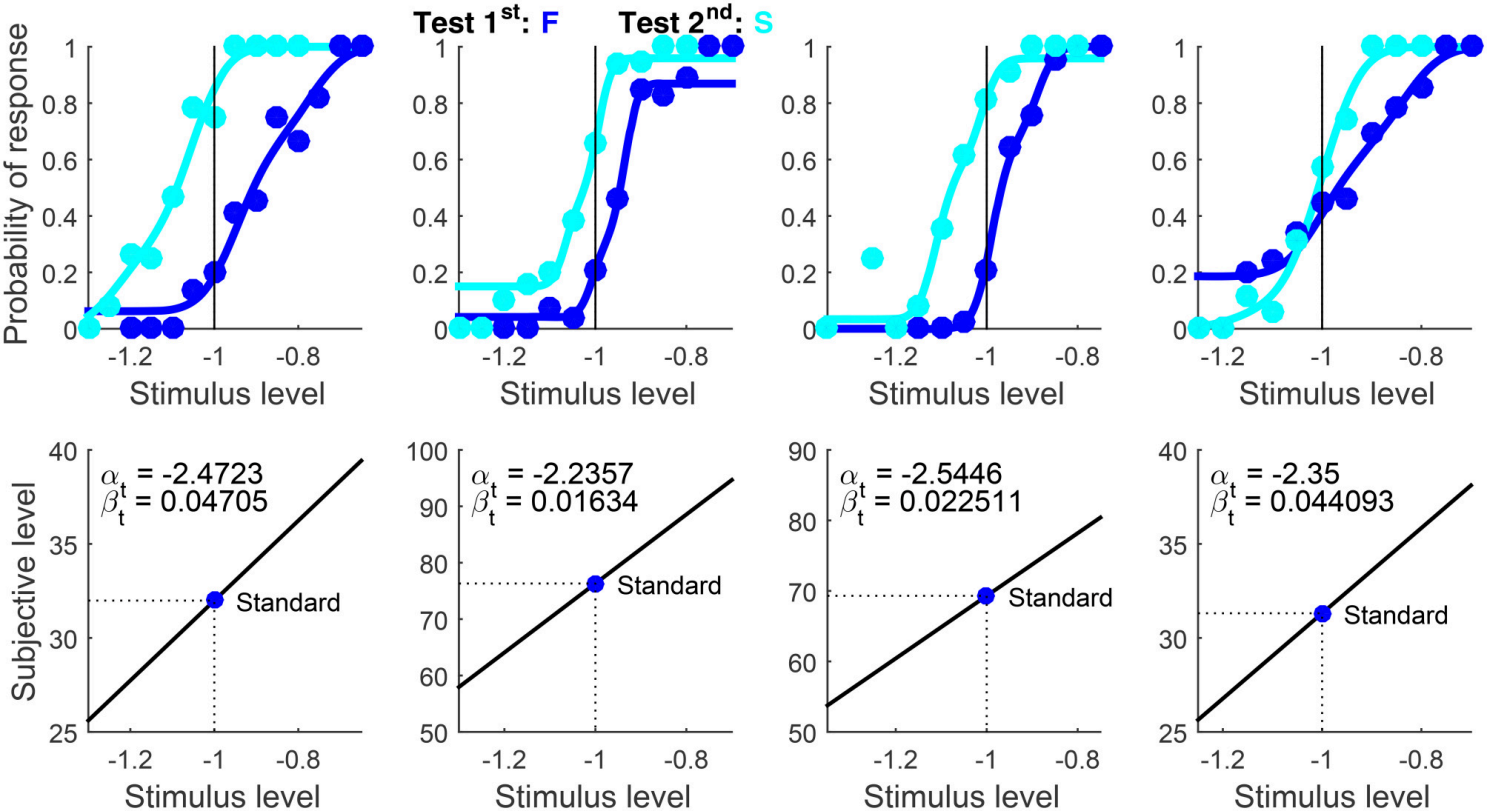
Rearranged graphical output for example 5, involving detection data collected from four observers (columns) with the classical binary response format in which observers are asked to guess when uncertain. Standard and test stimuli were identical except along the dimension of comparison and, hence, a common psychophysical function holds. Goodness-of-fit statistics did not reject the fitted model for any observer.

Results are shown in Figure 14, and recall that α_t_ (whose estimate hit the lower bound for the fourth observer) is unidentifiable in these conditions. A re-run setting Format = ‘ternary’ rendered identical plots (plus data points and curves pertaining to the other response categories) and nearly identical parameter estimates. This reveals again that the ternary model fits 2AFC data equally well, although the returned counts of free parameters and degrees of freedom and the *p*-values are incorrect for truly binary data.

As in the preceding example, enforcing the assumption that δ_1_ = δ_2_ (results not shown; script available as example 5bis in the Supplementary Material) produced nearly identical curves with different estimates of β_t_ (recall that the estimated α_t_ is arbitrary here), further attesting to the inescapable confound of decisional and bias parameters in 2AFC data and to the suitability of the ternary format to resolve this empirical issue. Also in this alternative fit, goodness-of-fit statistics did not reject the fitted model for any observer but output field 27 reported that the upper bound for width was hit, indicating that the data would be better fitted if δ_2_ > δ_1_ were allowed.

It is obvious that μ_t_ = μ_s_ when test and standard differ only along the dimension of comparison, as in this case: Test and standard are only experimental designations, but their sensory processing must be identical and reflect the characteristics of the (single) underlying psychophysical function. Then, whether or not μ_t_ = μ_s_ is not an experimental hypothesis in these conditions. Estimating parameters under the assumption that μ_t_ ≠ μ_s_ instead (by setting Type = ‘diff’) only allows some extra flexibility that nevertheless does not produce meaningfully different estimates. Readers can confirm this by re-running the scripts for examples 5 and 5bis after setting Type = ‘diff’.

### Example 6. visual discrimination of line length; 2AFC responses (U not allowed)

Data for this example come also from the study that provided data for example 3. Data from the same observers in an identical discrimination task that used instead the 2AFC format were presented in Table 1 of that paper. Overall, 100 trials were administered at each test level with each presentation order. The analysis fitted logistic functions to data from each presentation order satisfying theoretical constraints on their slopes and locations (see Figure 3 in García-Pérez and Alcalá-Quintana, 2011b). To fit the indecision model to these data, the script set Format = ‘2AFC’, Standard = 104, Type = ‘diff’ and, as in example 3, AlphaBounds = [−5 5].

**Figure 15:**
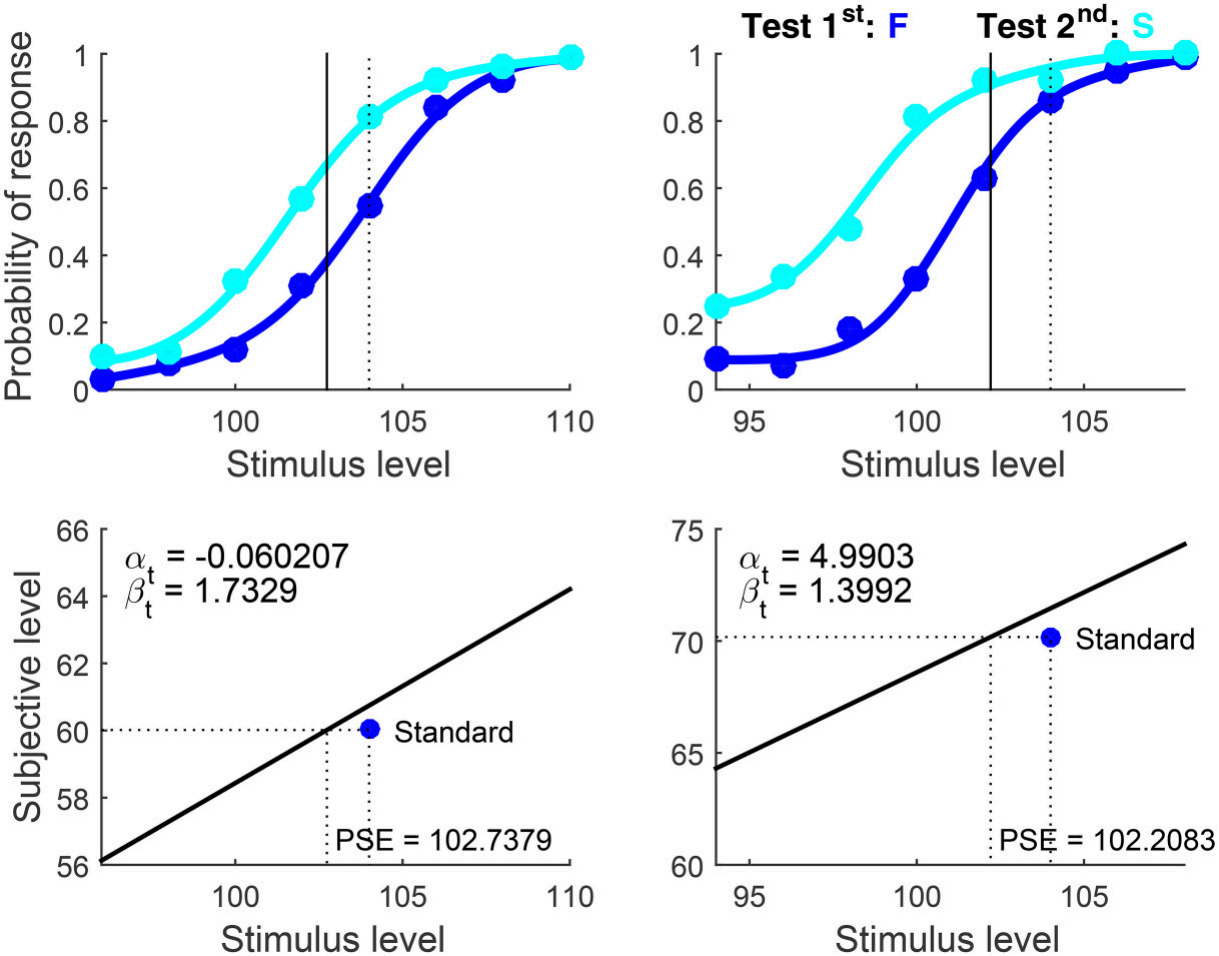
Rearranged graphical output for example 6, involving discrimination data collected from two observers (columns) with the classical binary response format in which observers are asked to guess when uncertain. Standard and test stimuli differed in orientation and, hence, data were fitted under the assumption that different psychophysical functions hold for test and standard. Goodness-of-fit statistics did not reject the fitted model for any observer.

The results are shown in Figure 15 in compact form, and recall that α_t_ is unidentifiable from these data. A re-run setting Format = ‘ternary’ reveals again that the unconstrained ternary model fits the data equally well, although miscounting the number of free parameters and with slightly different parameter estimates.

The data can also be nearly identically accounted for on the assumption that δ_1_ = δ_2_ (results not shown; script available as example 6bis in the Supplementary Material) but with different estimates of β_t_ and the anchor μ_s_(*x*_s_). This renders different estimates of the PSE also. Estimating parameters under this assumption did not reject the model for any observer but, again, output field 27 reported that the upper bound for width was hit so that the data would be better fitted if δ_2_ > δ_1_ were allowed.

One would expect that an attempt to fit these data with μ_t_ = μ_s_ will fail, given that length discrimination with lines of different orientation is well-known to involve μ_t_ ≠ μ_s_ (Armstrong and Marks, 1997). But this is not the case: Confound of decisional and bias parameters permits accounting for 2AFC discrimination data nearly identically with μ_t_ = μ_s_ and with μ_t_ ≠ μ_s_. Readers can confirm this by re-running the script for example 6 after setting Type = ‘same’. In contrast, re-running the script for example 6bis (which additionally enforces the assumption that δ_1_ = δ_2_) after setting Type = ‘same’ does fail to fit the data. Facing analogous results in a study aimed at determining whether or not μ_t_ = μ_s_ (i.e., in cases in which it is unclear whether or not the extra dimension on which test and standard differ has some perceptual effect), an experimenter will be unable to answer the question: μ_t_ = μ_s_ is tenable if one assumes δ_1_ ≠ δ_2_ but it is untenable if one assumes δ_1_ = δ_2_. As discussed in example 3 above, ternary data solve this indeterminacy: U responses inform of δ_1_ and δ_2_, allowing an unambiguous test of μ_t_ = μ_s_ against μ_t_ ≠ μ_s_.

### Example 7. auditory discrimination of frequency modulation rate; same–different responses

Data for this example come from a study that used the same–different format to assess auditory discrimination of frequency modulation rate (Umbach and Wickelmaier, 2014). Modulation rates ranged from 5.9 to 9.1 Hz in steps of 0.4 Hz, and data from three observers were collected for all pairs of modulations in both presentation orders. The study investigated the principle of regular minimality (Dzhafarov, 2002) and data had been analyzed as needed for that purpose (see Umbach and Wickelmaier, 2015). We selected for this example the subset of data involving the modulation rate at the center of the range (i.e., 7.5 Hz), which is the standard level in this analysis. The number of trials administered at each test level differed by design and ranged from 60 (at the test level furthest from the standard) to 150 (when test and standard had the same level) per presentation order.

The script set Format = ‘equality’ (see the usage documentation), Standard = 7.5 (i.e., the modulation rate of the standard tone), and, because test and standard only differed as to modulation rate, Type = ‘same’. The data were clearly collected at suprathreshold levels but it is not clear where the detection threshold for modulation rate may lie; hence, AlphaBounds was set with the general rules for suprathreshold discrimination (i.e., as in examples 2 and 5), yielding here the range between −0.5 and 2.7.

**Figure 16:**
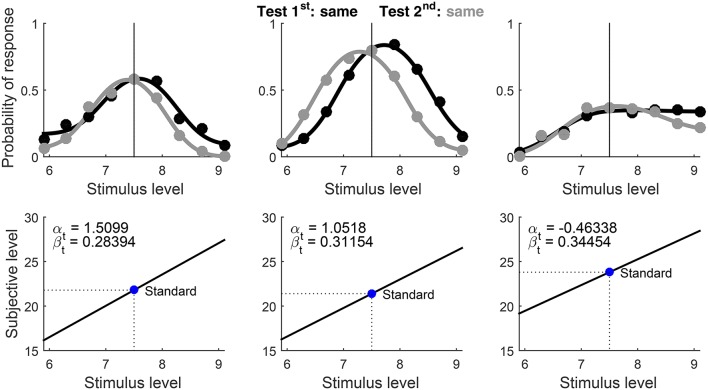
Rearranged graphical output for example 7, involving discrimination data collected from three observers (columns) with the same–different response format in which observers report whether the two stimuli are subjectively equal or different. Standard and test stimuli were identical except along the dimension of comparison and, hence, a common psychophysical function holds. Goodness-of-fit statistics did not reject the fitted model for any observer.

Results are shown in Figure 16, and note that only data and psychometric functions for “same” responses are plotted. Again, α_t_ is unidentifiable with these data. A re-run with Format = ‘ternary’ reveals that the unconstrained ternary model fits these data identically and with virtually identical parameter estimates (because decisional and bias parameters are not confounded here), but miscounting the number of free parameters.

Obviously, same–different data cannot be accounted for with δ_1_ = δ_2_, as this implies that “same” responses are never given (except, perhaps, as misreports). Then, same–different data also eliminate the ambiguity of 2AFC data in studies aimed at testing whether μ_t_ = μ_s_. In the current example, test and standard that do not differ except along the dimension of comparison imply μ_t_ = μ_s_ and, thus, re-running the script after setting Type = ‘diff’ is only expected to produce minimally different parameter estimates due to the extra flexibility but with the estimated PSE virtually at the standard level (i.e., *x*_PSE_ ≈ *x*_s_). This turns out to be true for the first and second observers and, depending on the matlab version that was used, also for the third. The latter outcome is understandable given the poor informative value of the data, collected at test levels that turned out to sample inadequately the psychometric functions for this observer. matlab versions rendering the unexpected *x*_PSE_ ≠ *x*_s_ fitted the data for this observer with −2log*L* = 2248.39 whereas those rendering the expected *x*_PSE_ ≈ *x*_s_ resulted in −2log*L* = 2248.36 instead, a (negligibly) smaller value indeed. Nearly identical −2log*L* indicates different solutions that fit the data equally well, a common outcome for poorly-informative data. Arguably, ternary data would have been useful in a case like this: Separate F and S responses (aggregated instead into “different” responses here) might have provided the extra information needed to constrain the fit with μ_t_ ≠ μ_s_ so that the optimal solution under this assumption involves parameter estimates analogous to those obtained in the fit under the assumption that μ_t_ = μ_s_.

## Conclusion

This paper has demonstrated that psychophysical data collected with a ternary response format in 2P tasks provide more accurate estimates of model parameters and performance measures than data collected instead with the binary 2AFC format or with the also binary same–different format. The ternary response format is also more natural than the 2AFC format with instructions to guess when uncertain, an admonition that only corrupts the data by mixing up authentic judgments and guesses. This mix-up is the main reason that 2AFC parameter estimates are less accurate, but it is also the reason that 2AFC data are uninformative when it comes to testing certain types of experimental hypotheses (as discussed in example 6). All things considered, use of the 2AFC format in psychophysical research is unadvisable.

Replacing the 2AFC response format with a ternary format for data collection is simple, but fitting psychometric functions to ternary data further separated by presentation order poses some challenges. This must also be done somewhat differently according to whether the data come from detection or discrimination tasks and, in the latter case, also according to whether the psychophysical functions for test and standard are assumed to be equal or different. The matlab and R routines (available as Supplementary Material) that were developed for our purposes in this paper fit psychometric functions from the indecision model implementing all of these options in a user-friendly way and, thus, they should help spread the use of the ternary format for dependable collection and interpretation of psychophysical data. An accompanying document also in the Supplementary Material provides complete usage information and discusses caveats and limitations.

## Author contributions

Both authors contributed equally to this work except that the matlab code was written by MAGP whereas the R code was written by RAQ.

### Conflict of interest statement

The authors declare that the research was conducted in the absence of any commercial or financial relationships that could be construed as a potential conflict of interest. The reviewer EP and handling Editor declared their shared affiliation, and the handling Editor states that the process nevertheless met the standards of a fair and objective review.

## Footnotes

^1^The meaning of the output flag in field 2 varies across versions of matlab due to the different algorithms implied. Users are referred to the help documentation for fmincon in their installation. This is also the only field that differs in the matlab and R versions of the routine. The R version gives instead output diagnostic information from optim, namely, the convergence code and the number of function evaluations, also arranged in vector form.
