# Supplementary material for: The Indecision Model of Psychophysical Performance in Dual-Presentation Tasks: Parameter Estimation and Comparative Analysis of Response Formats
Source: Front Psychol. 2017 Jul 12;8:1142. doi: 10.3389/fpsyg.2017.01142 (PMC5506217; doi:10.3389/fpsyg.2017.01142)
Supplement: Supplementary file 1 [file Presentation1.zip › Usage_Documentation.pdf]

The indecision model of psychophysical performance in dual-presentation tasks: Parameter estimation and comparative analysis of response formats

Frontiers in Psychology, 8:1142

Usage Documentation

Miguel A. García-Pérez & Rocío Alcalá-Quintana
Departamento de Metodología, Facultad de Psicología, Universidad Complutense,
Campus de Somosaguas, 28223 Madrid, Spain

miguel@psi.ucm.es ralcala@psi.ucm.es

Introduction

This document presents usage notes for the MATLAB and R routines that fit psychometric functions from the indecision model to ternary dual-presentation (2P) data from detection or discrimination tasks. Provided that data are still separated across presentation orders, the routines can also fit the model to 2P data collected with the classical binary two-alternative forced-choice (2AFC) format or with the also binary same–different format, although the use of these two response formats is not advisable given the inferior accuracy of the parameter estimates that they provide (as demonstrated via simulation in the paper). The routines fit the model with a choice of assumptions about same or different psychophysical functions for test and standard stimuli (for discrimination data) and with a choice of error model, including also the option to explore all error models and return parameter estimates for the error model that best fits the data by a criterion of choice.

Our focus in this document is a thorough description of the calling statements and output of the routines, a description of how they were used to estimate parameters in the simulation study presented in the paper, and a presentation of usage notes and recommendations.

Calling statements and arguments

Exhibit 1 in the paper showed a sample script illustrating the use of the MATLAB version of the routine. Exhibit S1 here shows an analogous script that uses the R version instead. The calling statement is shown as the last line in each exhibit and, in both cases, the arguments are as follows:

`Data:` $7 \times N$ matrix with data arranged so that the first row lists the N test levels at which data were collected, rows 2–4 list the counts of F, U, and S responses at each level when the test was presented first, and rows 5–7 list the counts of F, U, and S responses at each level when the test was second. If no data were collected at one or more test levels under one of the presentation orders, the corresponding entries in the data array must be filled up with 0’s (as seen in

the exhibits for test-second presentations at the first test level). If `Format = '2AFC'` (see below), rows 3 and 6 must be filled up with zeros (as no U responses exist); if `Format = 'equality'`, rows 2 and 5 must be filled up with zeros (i.e., "same" responses are stored in the place of U responses and "different" responses are stored in the place of S responses).

```
source(fit_TIM_2P)
Data <- rbind(c(243,245,247,249,251,253,255,257,259,261),
              c(  0,  3,  3, 10, 22, 17, 16, 13,  8,  7),
              c(  0,  1,  6,  6, 10,  0,  1,  1,  0,  0),
              c(  8, 15, 18, 24,  2,  1,  0,  0,  0,  0),
              c(  0,  6,  6, 14, 17, 23, 28, 17,  6,  0),
              c(  0,  0,  0,  0,  0,  2,  5,  6,  4,  0),
              c(  0,  0,  0,  0,  1,  1,  7, 19, 17, 13))
Standard <- 251.86
AlphaBounds <- c(207,225); BetaBounds <- c(0.01,4)
Delta1Bounds <- c(-6,2); WidthBounds <- c(0,10)
AlphaStart <- c(216); BetaStart <- c(2,3); Delta1Start <- c(-3,-1,1)
WidthStart <- c(4); EpsStart <- c(0.03); KappaStart <- c(0.5)
Model <- 'best'; Criterion <- 'LogL'
Type <- 'diff'; Disp <- TRUE; Plot <- TRUE; Format <- 'ternary'
o <- fit_TIM_2P(Data, Standard, Format,
                AlphaBounds, BetaBounds, Delta1Bounds, WidthBounds,
                AlphaStart, BetaStart, Delta1Start, WidthStart,
                EpsStart, KappaStart, Model, Criterion, Type, Plot, Disp)
```

Exhibit S1. R script to fit the indecision model.

`Standard:` A real scalar indicating the level of the standard stimulus. For detection data, set `Standard = -Inf` even if the null standard has a different value in the units in which it is measured (e.g., 0 in a luminance-detection task). This is the implicit manner in which discrimination and detection are distinguished, making the function implement the applicable constraints.

`Format:` A string (case insensitive) indicating whether the data were collected with the ternary response format (`'ternary'`), with the 2AFC format (`'2AFC'`), or with the same–different format (`'equality'`).

`AlphaBounds:` A 1×2 vector stating (different) lower and upper bounds for α_t (reals). Criteria for the choice of these and subsequent bounds are discussed below.

`BetaBounds:` A 1×2 vector stating (different) lower and upper bounds for β_t (positive reals).

`Delta1Bounds:` A 1×2 vector stating (different) lower and upper bounds for δ_1 (reals).

`WidthBounds:` A 1×2 vector stating (different) lower and upper bounds for the distance between δ_1 and δ_2 (non-negative reals). To fit 2AFC data (i.e., when `Format = '2AFC'`) enforcing the assumption that $\delta_1 = \delta_2$, set `WidthBounds = [0 0]`. This is the only case in which equal lower and upper bounds are permitted (but both valued at 0).

`AlphaStart:` Starting value(s) for α_t (reals). It can be a scalar (to set a single starting value) or a row vector (to set several of them) with value(s) within `AlphaBounds`.

`BetaStart:` Starting value(s) for β_t (positive reals). It can be a scalar or a row vector with value(s) within `BetaBounds`.

`Delta1Start`: Starting value(s) for δ_1 (reals). It can be a scalar or a row vector with value(s) within `Delta1Bounds`.

`WidthStart`: Starting value(s) for the amount by which δ_2 exceeds δ_1 (non-negative reals). It can be a scalar or a row vector with value(s) within `WidthBounds`. When `WidthBounds = [0 0]` is permitted (see above), `WidthStart = 0` is naturally the only option.

`EpsStart`: Starting value(s) for all error parameters (ε_F, ε_U, and ε_S for each presentation order), in the interval [0, 1]. It can be a scalar or a row vector.

`KappaStart`: Starting value(s) for all bias parameters (the κs for each presentation order), in the interval [0, 1]. It can be a scalar or a row vector.

`Model`: Choice of model, according to codes in Fig. 3b of the paper. To fit different models for each presentation order, this must be a 1×2 vector whose components are the model to be used when the test was presented first and second, respectively. Each of them must be an integer between 0 and 7, inclusive. To use the same model in both cases, a scalar suffices (e.g., set `Model = 1` instead of `Model = [1 1]`). To find the best-fitting model by the criterion of choice (see below), set `Model = 'best'` (case insensitive). When `Format = '2AFC'`, only error models 1, 2, 4, and 6 are allowed because ε_U must be in the fitted model with a fixed value of unity (see Fig. 4a in the paper). Analogously, when `Format = 'equality'`, only models 1, 2, 3, and 5 are allowed because ε_F must be in the fitted model with a fixed value of unity (see Fig. 4b in the paper). Setting `Model = 'best'` enforces these restrictions on models for 2AFC and same–different data.

`Criterion`: A string (case insensitive) indicating the criterion to select the best-fitting model when `Model = 'best'`. Options are the Bayesian Information Criterion (`'BIC'`) and the log-likelihood of the data under the model (`'LogL'`). This argument is immaterial when model selection is not involved (i.e., when `Model` is *not* set to `'best'`).

`Type`: A string (case insensitive) indicating whether data should be fitted assuming the same (`'same'`) or different (`'diff'`) psychophysical functions for standard and test (i.e., $\mu_t = \mu_s$ or $\mu_t \neq \mu_s$, respectively). For detection data (i.e., when `Standard = -Inf`), user input is ignored and `Type = 'same'` enforced.

`Plot`: A logical scalar. If true, plots like those in Figs. 6 and 7 in the paper are created.

`Disp`: A logical scalar. If true, messages are issued to the console as operation progresses. If a single model was selected, a message is issued for each of the multidimensional starting points tried out; if `Model = 'best'`, a single message is issued for each candidate model which refers to the optimal solution found across starting points.

Output

Graphical output (if `Plot = true`) for the sample script in Exhibit 1 of the paper was presented and discussed there and will not be reproduced here. The output structure returned by that script is reproduced in Fig. S1 here for self-containedness.

Field	Value
Problem	'Fit of the indecision model to dual-p...
fmincon_output	[2 52 975]
Data	7x10 double
Format	'ternary (respond first, second, or u...
Standard	251.8600
UserModel	'best'
FittedModel	[1 2]
Criterion	'LogL'
Type	'diff'
NumFreeParameters	[15 14]
NumCells	57
NumExpBelow5	33
NumExpBelow5_ObsAbove0	12
NumExpBelow1	25
NumExpBelow1_ObsAbove0	5
DegreesOfFreedom	[23 24]
ChiSquareStatistic	19.9562
ChiSquareP_value	[0.6446 0.6993]
LikelihoodRatioStatistic	24.2604
LikelihoodRatioP_value	[0.3895 0.4468]
BIC	[519.4296 516.4852]
Neg2LogL	475.2630
AlphaBounds	[207 225]
BetaBounds	[0.0100 4]
Delta1Bounds	[-6 2]
WidthBounds	[0 10]
BoundariesReached	' none '
Alpha_t	219.7193
Beta_t	1.4619
Mu_s_at_StandardLevel	23.3176
Mu_t_at_StandardLevel	22.6786
Delta_1	1.7400
Delta_2	3.5526
Epsilon_F_1	0.0343
Epsilon_U_1	0.4704
Epsilon_S_1	0.1008
Kappa_FintoU_1	1
Kappa_FintoS_1	0
Kappa_UintoF_1	0
Kappa_UintoS_1	1
Kappa_SintoF_1	1
Kappa_SintoU_1	0
Epsilon_F_2	0.0180
Epsilon_U_2	0.6433
Epsilon_S_2	NaN
Kappa_FintoU_2	0
Kappa_FintoS_2	1
Kappa_UintoF_2	0.6731
Kappa_UintoS_2	0.3269
Kappa_SintoF_2	NaN
Kappa_SintoU_2	NaN
PSE	252.7941
DL	1.3945
Threshold_84	'notapplicable'

Figure S1. Output structure from the MATLAB script in Exhibit 1 of the paper.

Recall that the output information includes a label for the problem (field 1), diagnostic codes from `fmincon` (or `optim` in the R version) the data, the response format, and the standard level (fields 3–5), the user-selected error model and the model for which parameters are returned (fields

6 and 7), the criterion selected to search for the best-fitting model, if applicable (field 8), the type of fit regarding psychophysical functions for test and standard (field 9), the number of free parameters in the fitted model (field 10), the total number of cells for goodness-of-fit tests, the number of cells in which expected frequencies were smaller than 5 and the number of those cells in which observed frequencies were non-null (fields 11–13), the number of cells in which expected frequencies were smaller than 1 and the number of those cells in which observed frequencies were non-null (fields 14 and 15), the degrees of freedom, values, and *p*-values of Pearson's chi-square (X^2) and the likelihood-ratio (G^2) goodness-of-fit statistics (fields 16–20), the BIC of the fitted model (field 21), the −2Log$L$ of the data under the fitted model (field 22), the user-defined content of `AlphaBounds`, `BetaBounds`, `DeltaBounds`, and `WidthBounds` (fields 23–26), a statement indicating which boundaries were reached, if any (field 27), estimates for α_t and β_t (fields 28 and 29), the estimated anchor $\mu_s(x_s)$ and the ordinate of μ_t at $x = x_s$ (fields 30 and 31), estimates of δ_1 and δ_2 (fields 32 and 33), estimates of the ε and κ parameters when the test was presented first (fields 34–42) and second (fields 43–51), and performance measures (PSE, DL, and detection threshold) as applicable (fields 52–54). Comments regarding these fields are given next.

If `UserModel` (field 6) is a specific model selected by the user, `Criterion` (field 8) is listed as `'not applicable'` regardless of user input. `NumFreeParameters` (field 10) is the number that applies to the error model reported in field 7 (`FittedModel`), taking into account that some error parameters are not free for 2AFC or same–different data (see Fig. 4 in the paper), and also when 2AFC data are additionally fitted under the assumption that $\delta_1 = \delta_2$. We should stress again that α_t is *not* a free parameter in suprathreshold discrimination, but this is indiscernible from the data. Thus, for discrimination data field 10 always lists two values, as in this example. Users must take the first value if the standard was near the detection threshold, when α_t is a free parameter, and the second value if the standard was suprathreshold, when α_t is not a free parameter. No such choice exists for detection data (i.e., when `Standard = -Inf`): Field 10 lists a single value instead because α_t is always a free parameter for detection data. In this example, which involves suprathreshold discrimination, the second value in field 10 applies and the 14 free parameters are β_t, the anchor $\mu_s(x_s)$ (given that different psychophysical functions were stated to hold for standard and test), δ_1 and δ_2, three ε's and the three associated κ's for trials in which the test was first (given that error model 1 was fitted to those data), and two ε's and the two associated κ's for trials in which the test was second (given that error model 2 was fitted to those data).

`NumCells` (field 11) reports the effective number of cells, which may be smaller than the number of apparent cells in `Data`, as is the case here. Note that `Data` contained 0's for test-second presentations at the lowest test level, indicating that no trials had been presented with those characteristics. All such cases (at one or the other presentation order) are removed from the count of cells, yielding 3×10 + 3×9 = 57 cells here. Each test level for which there are data at some presentation order contributes two degrees of freedom under the ternary format (but only one under 2AFC or same–different formats). In this case, then, data contribute 2×10 + 2×9 = 38 degrees of freedom but each free parameter subtracts one, yielding the 38 − 14 = 24 degrees of

freedom reported as the second value in field 16 (`DegreesOfFreedom`), which also lists two values for discrimination data for the reason discussed regarding `NumFreeParameters` (field 10). (When α_t is a free parameter with discrimination data, users must take the first value instead; for detection data, where α_t is always a free parameter, field 16 lists a single value.) If the difference(s) turn up non-positive, field 16 reports those value(s) for an indication of the number of additional test levels that would solve the problem, but `NaN` is reported in fields 18 and 20 instead of the *p*-values of the goodness-of-fit statistics whose values are still reported in fields 17 and 19. Two *p*-values are also listed in fields 18 and 20 for discrimination data because this value depends on the number of free parameters; goodness-of-fit statistics in fields 17 and 19 are always unique. Note that goodness-of-fit statistics do not reject the model here (second values in fields 18 and 20, on consideration that α_t is not a free parameter here). Two values are also reported for the BIC (field 21) for discrimination data (but only one for detection data), again because its value depends on the number of free parameters; the applicable value (first or second) is chosen with the same criterion as for the remaining dual-valued fields (i.e., `NumFreeParameters`, `DegreesOfFreedom`, `ChiSquareP_value`, and `LilelihoodRatioP_value`).

Ideally, field 27 (`BoundariesReached`) confirms that the optimization algorithm did not attempt to surpass the bounds defined for free parameters (reproduced from user input in fields 23–26 for convenience), as in this example. In other cases, this field lists the boundaries that were hit (e.g., `'lower_Beta upper_Delta1'`), which is always accompanied by parameter estimates at those boundaries. A re-run with expanded bounds should often be attempted (see below). Yet, when α_t is not a free parameter (hence, it is not identifiable and its value does not affect the fit), messages indicating that one of its bounds was reached should be ignored.

The value and status as a parameter of the anchor $\mu_s(x_s)$ (field 30) varies across cases. For detection data, the anchor valued at 0 is not a free parameter. For discrimination data with identical psychophysical functions for test and standard, the anchor is not a free parameter either and its value is determined by the estimates of α_t and β_t. The anchor is a free parameter (and is counted as such) only for discrimination data with different psychophysical functions for test and standard, as in this example. In these cases, its value will generally differ from the value of $\mu_t(x_s)$ in field 31.

Fields 34–51 list all the ε and κ parameters in Eqs. 3 of the paper, including the κ's that are not free parameters (i.e., the two adjacent κ's in each pair add up to unity). However, when the fitted model excludes one or more of the ε parameters, those and their associated κ's are listed as `NaN` (or `NA` in R), as in this example. Error parameters excluded from the model are actually valued at 0 but they are returned as `NaN` to differentiate such cases from those in which error parameters intentionally included in the model were estimated to be 0. For 2AFC data (i.e., when `Format = '2AFC'`) and for same–different data (i.e., when `Format = 'equality'`), some ε's and κ's will be returned as 0 or 1 without them being free parameters (see Fig. 4 in the paper).

All of the above needs qualification when 2AFC data are fitted by enforcing the assumption that $\delta_1 = \delta_2$ (i.e., when `WidthBounds = [0 0]`, as described above). In this case, the number of free parameters is reduced by three (i.e., δ_2, $\kappa_{U\text{–}F,1}$, and $\kappa_{U\text{–}F,2}$ are not free parameters in this case),

which is taken into account in the reported number of free parameters, degrees of freedom, *p*-values, and BIC. Accordingly, `Delta_2` (field 33) , `Epsilon_U_1` (field 35) , `Kappa_UintoF_1` (field 39) , `Kappa_UintoS_1` (field 40) , `Epsilon_U_2` (field 44) , `Kappa_UintoF_2` (field 48) , and `Kappa_UintoS_2` (field 49) are all reported as `NaN` (or `NA` in R). Quite often, fitting 2AFC data under this assumption results in `BoundariesReached` (field 27) reporting `'upper_Width'` which suggests that a better fit will be obtained if $\delta_1 \neq \delta_2$ were allowed.

Finally, performance measures in fields 52–54 are computed as defined in the paper, if applicable. For discrimination data, the PSE and the DL are always reported and the former will match the standard level if the same psychophysical functions were assumed to hold for test and standard. For detection data, only the detection threshold is reported.

Settings used in the simulation study reported in the paper

To fit the simulated data presented in the paper, scripts were written as follows. Parameter bounds were defined identically in all seven scenarios: [−3.5, −1.5] for α_t, [0.01, 0.5] for β_t, [−6, 0] for δ_1, and [2, 10] for the width $\delta_2 - \delta_1$. Starting values were also defined identically in all cases: −2.5 for α_t, 0.1 and 0.2 for β_t, −3 and −2 for δ_1, 6 for the width $\delta_2 - \delta_1$, 0.03 for the ε's, and 0.5 for the κ's (the latter two are irrelevant when error model 0 was fitted). These common bounds and starting values attest to the robustness of the routines to recover parameters with arbitrarily different true values. Data generated with response errors were fitted with error model 1; data generated without response errors were fitted with the appropriate error model: model 0 for ternary data, model 6 for 2AFC data, and model 5 for same–different data.

Results for data generated without response errors were presented in Fig. 5 of the paper; Fig. S2 here displays the results for data generated with response errors. Note that, in general, parameter recovery is poorer across the board in this case, but ternary data continue to provide more accurate parameter estimates than 2AFC or same–different data.

Differences between MATLAB and R implementations

All the results presented in the paper were obtained with the MATLAB version of the routine, which relies on the robust and stable algorithms implemented in the built-in function `fmincon`. As a result, the MATLAB routine proved highly insensitive to starting values set within the guidelines discussed below and it is unlikely that other starting values will ever have to be tried out in order to arrive at a good solution.

Running the accompanying R scripts for examples 1–7 will reveal that the R function `optim` does not always arrive at the solutions shown in Figs. 9–16 of the paper despite the identical settings for bounds and starting values. In fact, a thorough comparison across all error models (not just the case `Model = 1` used in those examples) and sets of starting values for these and other data sets showed that the output −2Log*L* under R was rarely minimally smaller (and often meaningfully larger) than it was under MATLAB with identical settings for the same data. The R version was also much more sensitive to starting values than its MATLAB counterpart. A further

indication of fundamental differences in the internal workings of `fmincon` (in MATLAB) and `optim` (in R) is clearly apparent when `Model = 'best'`. In such cases, the MATLAB function invariably (and naturally) returns the smallest value of −2Log*L* for model (1, 1), although this often involves some error parameters estimated at 0. Subsequently along the analysis of all error models, an alternative model that fixes those error parameters to 0 also returns the same value of −2Log*L*. (In such cases, the routines return the solution with structural as opposed to estimated null error parameters.) In contrast, the R function rarely returns the lowest −2Log*L* for model (1, 1), indicating that `optim` was incapable of finding for this model a better solution with null error parameters where other models fix them to 0.

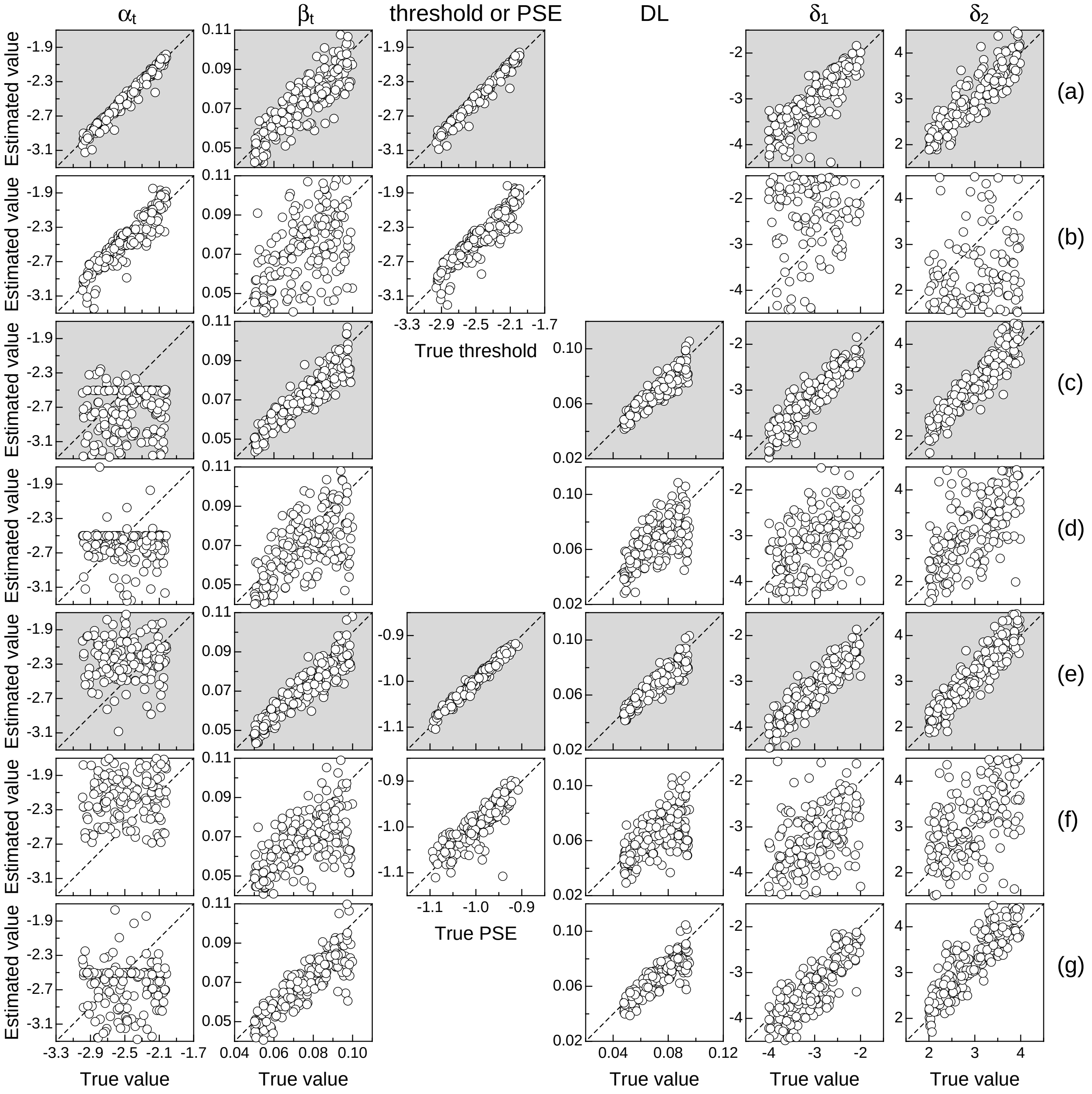

Figure S2. Simulation results for data generated with response errors.

Still another indication of the limited capabilities of the R function `optim` is apparent upon running the scripts for examples 4–7 (which involve 2AFC or same–different data) after setting `Format = 'ternary'`. As discussed in the paper, an analogous change does not affect the output of the MATLAB function, but the R function was rarely capable of returning a solution that estimates the appropriate error and bias parameters to their known fixed values (see the tree diagrams in Fig. 4 of the paper). Instead, `optim` often stops and returns with a non-fitting solution in which these parameters are still far from their known values.

Why `optim` cannot always return a proper solution is unclear, but some of the difficulties accompanying it are well known (see, e.g., Nash, 2014). It is nevertheless true that, with patience and dedication, starting values can be found that make the R version of the routine arrive at the optimal solution that the MATLAB version finds immediately. Users should keep this in mind if they are initially uninclined toward one or the other environment.

Recommendations for the choice of bounds and starting values

Examples 1–7 in the paper described criteria for the choice of bounds for α_t in detection and discrimination tasks, and these will not be reproduced here. However, general guidelines for the choice of the remaining bounds should be given. Note that the guiding principle is always to obtain the best-fitting solution for the data on hand. A judicious choice of these bounds thus helps the routine to arrive at the solution that was there to be found (if it was).

Consider `Delta1Bounds` first. Except for extreme cases of decisional bias, δ_1 will be on the negative side of the domain of *D* (see Fig. 1a in the paper). Furthermore, since *D* is normally distributed with variance 2 irrespective of the range and scale of stimulus levels, δ_1 will rarely be more than 3–4 standard deviations below the null value of *D*. This consideration leads to the invariable bounds at −6 and 2 used in all the examples, which should be adequate in other cases.

Reasonable bounds for $\delta_2 - \delta_1$ (i.e., `WidthBounds`) come from analogous considerations on the normal metric of decision space. The minimum width is 0, which will occur for observers who are never undecided; at the other end, a realistically large value is 10, which will occur for observers who are always undecided when test and standard elicit the same average sensory effects. These considerations lead also to the bounds used in all the examples, and they should also be adequate in most other cases.

Defining suitable bounds for β_t requires consideration of the range of test levels. As seen in Fig. 1b of the paper, $1/\beta_t$ determines the slope of the mapping of stimulus levels (in their own metric) onto perceived levels (always in the metric of a normal distribution with variance 2). Data always show that the psychometric functions for one of the presentation orders display non-asymptotic behavior between stimulus levels x_{inf} and x_{sup} (the range for the other presentation order will be similar, but perhaps displaced). Such range may be narrower than the range of test levels at which data were collected (as in the left column of Fig. 10 in the paper), roughly coincident with it (as in the right column of Fig. 10 in the paper), or broader (as in the right column of Fig. 11 in the

paper). Given the normal metric of perceived levels, a rule of thumb sets the lower bound for β_t at $(x_{sup} - x_{inf})/100$ and the upper bound at $(x_{sup} - x_{inf})/5$. The results obtained with `BetaBounds = [0.01 4]` in all of our examples suggests that setting bounds with this rule is not crucial, but this is only because $x_{sup} - x_{inf}$ varied between 1 and 20 in all of them. When $x_{sup} - x_{inf}$ is larger or smaller than these figures, bounds for β_t would rather be set with the expressions just given.

Setting suitable bounds for all parameters at first is not crucial, as the routine reports which bounds were reached and should be adjusted in a re-run. For an illustrative example regarding the setting of bounds for β_t, consider the artificial, suprathreshold 2AFC discrimination data created by Bausenhart et al. (2012) to demonstrate their software. The data (see the left panel of Fig. S3) indicate that $x_{sup} - x_{inf} \approx 80$. As discussed in the preceding paragraph, the lower and upper bounds for β_t should then be set at 0.8 and 16, respectively. Ignoring this and calling the function with `BetaBounds = [0.01 4]` as in all our previous examples results in a poor fit (whether judged by eye or via the reported p-values) that is also accompanied by β_t estimated at the upper bound of 4 (emphasized by the message reported under `BoundariesReached` in the output structure or list).

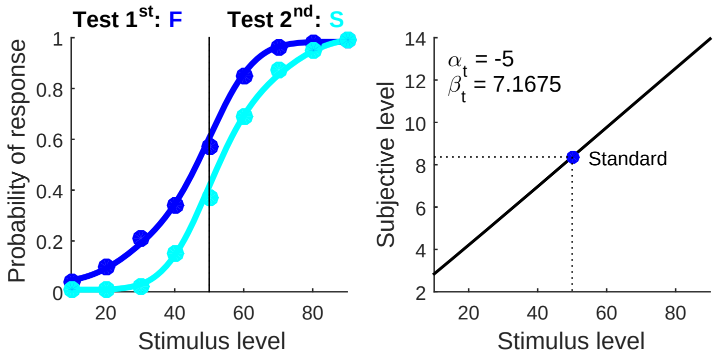

Figure S3. Rearranged graphical output for example 8 (script available in the Supplemental Material), for the artificial 2AFC discrimination data created by Bausenhart et al. (2012). Sensory and decisional processes were not modeled and data were simply generated by a binomial process on arbitrary psychometric functions that differed in location and slope across presentation orders. Goodness-of-fit statistics did not reject the fitted model.

This result is understandable on the basis of what `BetaBounds` should have been by the rule discussed above. A re-run with `BetaBounds = [0.8 16]` instead (and with no additional changes in the bounds for other parameters or the starting values for any parameter) produces the results shown in compact form in Fig. S3, which makes two additional points. One is that our software has the same functionality as that of Bausenhart et al. (2012) for 2AFC discrimination data; the other is that the indecision model can account for what Ulrich and Vorberg (2009) called Type-A and Type-B order effects (i.e., differences in the location or in the slope, respectively, of the psychometric functions for each presentation order), something that was already clear in Figs. 14 and 15 in the paper.

Another important aspect of usage is the choice of starting values for each parameter. Ideally, the likelihood function for the data is well behaved and the global optimum is reached regardless

of the starting point in parameter space. More realistically, and particularly when the data are noisy, the likelihood function will have multiple local optima to which the parameter-estimation algorithm may be differentially driven depending on starting point. In our experience with these routines (and as our examples attest to), a single starting value generally suffices for `AlphaStart`, `WidthStart`, `EpsStart`, and `KappaStart`, set as shown in those examples. Occasionally, setting `WidthStart` to that single value may prove inadequate and, thus, providing two (e.g., 1 and 4) may be useful. In contrast, `BetaStart` and `Delta1Start` should include two or three values (as in our examples) because these parameters seem to be maximally responsible for local optima.

The appropriateness of the chosen starting values can be assessed by setting `Disp` to true and setting `Model` to 1: The final value of $-2\text{Log}L$ will be displayed for each individual starting point in multidimensional space and, hence, nearly identical values across starting points will indicate that the choice is immaterial whereas broadly diverse values will indicate that some starting values were inadequate. Yet, even in the latter case, no further action may be needed if the best solution across all starting points is adequate, something that can be assessed in the output plots (if `Plot` was set to true) and via goodness-of-fit measures reported in the output structure or list.

Recommendations for a re-run with alternative settings

Provided that parameter bounds are defined as discussed in the preceding section, the most common (though infrequent) cause of a failure to fit the data is an unfortunate choice of starting values, particularly for β_t and for the width $\delta_2 - \delta_1$. Inadequate starting values for these parameters may place starting points in multidimensional space within a region where the likelihood function is flat or nearly flat, resulting in an improper early return. Also, inadequate starting values may drive the search algorithm toward boundaries in parameter space from which no escape seems feasible. Rather often, the proper action is to change starting values instead of expanding the bounds. Although execution time is very short per starting point in multidimensional space, the search for appropriate starting values for the parameters (should the necessity arise) is more efficiently accomplished by replacing starting values than by adding more of them.

Choice of an error model

Fitting error model 1 is the recommended option in the very first analysis of data, which should aim at checking out the adequacy of bounds and starting values for each parameter. Setting true the logical arguments `Plot` and `Disp` is useful at this stage to gather quantitative and graphical evidence of the appropriateness of those choices.

Fitting error model 1 may produce null or near-null estimates for one or more of the error parameters (ε_F, ε_U, and ε_S for each presentation order) and, hence, in a statistical misfit of the data due to the inclusion of unnecessary parameters in the model. A re-run to fit the simplified error model suggested by the initial results for error model 1 allows a test between the two nested models, using the BIC or the G^2 statistics returned in each case (for details on how to conduct the test based on G^2, see Collett, 2003, Sections 3.9–3.11). It should also be kept in mind that the BIC

may select a model that the G^2 statistic rejects (see García-Pérez, 2017; García-Pérez & Alcalá-Quintana, 2012). An alternative one-shot approach involves setting `Model = 'best'` (choosing also `'BIC'` or `'LogL'` as `Criterion`), but this can be time-consuming in combination with multiple starting values for each parameter and, thus, unadvisable until the appropriateness of bounds and starting values has been established: Use of this option implies that 8 × 8 = 64 error models must be evaluated for ternary data (or 4 × 4 = 16 models for 2AFC or same–different data), increasing execution time meaningfully.

Protection against misuse

The functions check for invalid input in all of the arguments, whenever this can be unequivocally identified. If invalid input is detected the functions return immediately, issuing a customized error message to the console. The content of that message should be sufficient for users to fix the problem and re-run. Undetectable invalid input (e.g., inadequate arrangement of responses in the data matrix) will have unpredictable consequences.

It should be noted that arguments `AlphaBounds`, `BetaBounds`, or `Delta1Bounds` whose two components are equal are invalid, as the optimization algorithm requires non-empty search ranges for all parameters. The same holds for `WidthBounds`, with the only exception that `WidthBounds = [0 0]` is permitted when `Format = '2AFC'`, which triggers a tailored procedure to fit 2AFC data under the assumption that $\delta_1 = \delta_2$.

Limitations

All the MATLAB results were obtained with version 8.4.0.150421 (release 2014b), but we checked that versions 8.2.0.701 (release 2013b) and 9.0.0.341360 (release 2016a) in 32-bit or 64-bit Windows systems do not produce meaningfully different results (if at all). The R function was tested with version 3.3.1 (released 2016-06-21). We do not expect the functions to perform differently under newer versions of MATLAB or R unless the built-in functions `fmincon` or `optim` change in some meaningful respect.

Users must be aware that not all data sets are well suited for fitting psychometric functions and that these routines (or others, for that matter) cannot make up for lack of information in the data. The most common form of data inadequacy is when stimulus levels do not probe the underlying psychometric functions in the relevant region, when the number of trials per level is insufficient to obtain dependable estimates of the proportions of responses of each type at each level under each presentation order, or when performance seems haphazard, is too noisy, or is uninformative (as discussed regarding S responses to test-second presentations in the center column of Fig. 12 of the paper, and also regarding “same” responses in the right column of Fig. 16 of the paper). Parameter estimation and hypothesis testing (e.g., a test between $\mu_t = \mu_s$ and $\mu_t \neq \mu_s$) will be compromised in such cases, although nothing but collecting more informative data can be done in this respect.

Finally, the three parametric assumptions incorporated in the routines should be stressed, and potential replacements discussed. The first of these assumptions is the form of the psychophysical

function in Eq. 2 of the paper. If this functional form turns out theoretically inadequate in some context, it can be straightforwardly replaced with any other suitable function (provided that the replacement also has two parameters), as all computations involving the psychophysical function or its inverse are handled in a separate function within the routine.

The second parametric assumption is the normal distribution of sensory effects. When the decision variable *D* is instead defined as the difference between two non-normally distributed variables (see, e.g., García-Pérez & Peli, 2014), Eqs. 3 in the paper would refer instead to the corresponding cumulative distribution, which should also be used in the computation of performance measures (PSE, DL, and detection threshold). Again, the routine can be easily amended to cope with such cases by replacing calls to the cumulative normal distribution or its inverse with calls to the replacement distribution.

The third and final parametric assumption is that the variance of sensory effects is constant and independent of stimulus level (the so-called equal-variance assumption). In cases such as discrimination of temporal duration, the standard deviation of perceived duration increases with chronometric duration and the model then needs another parameter to capture this relation (see García-Pérez, 2014). Amending these routines to cover such cases requires additional programming, but alternative action is possible. The simplest approach is to express stimulus levels as the logarithm of the nominal levels, which is indeed the approach via which an identical problem is tackled in visual contrast perception. We have tested the adequacy of this approach with some data on duration discrimination and the results seem satisfactory. However, users are warned that treating violations of the equal-variance assumption by log-transforming stimulus levels may not be a suitable (or sensible) solution in all cases.

Acknowledgments

This research was supported by grant PSI2015-67162-P from Ministerio de Economía y Competitividad (Spain).

References

Bausenhart, K. M., Dyjas, O., Vorberg, D., & Ulrich, R. (2012). Estimating discrimination performance in two-alternative forced choice tasks: Routines for MATLAB and R. *Behavior Research Methods*, *44*, 1157–1174. doi:10.3758/s13428-012-0207-z

Collett, D. (2003). *Modelling Binary Data* (2nd edition). Boca Raton, FL: CRC Press.

García-Pérez, M. A. (2014). Does time ever fly or slow down? The difficult interpretation of psychophysical data on time perception. *Frontiers in Human Neuroscience*, *8*:415. doi:10.3389/fnhum.2914.00415

García-Pérez, M. A. (2017). Thou shalt not bear false witness against null hypothesis significance testing. *Educational and Psychological Measurement*, in press. doi:10.1177/0013164416668232

García-Pérez, M. A., & Alcalá-Quintana, R. (2012). On the discrepant results in synchrony

judgment and temporal-order judgment tasks: A quantitative model. *Psychonomic Bulletin & Review*, *19*, 820–846. doi:10.3758/s13423-012-0278-y

García-Pérez, M. A., & Peli, E. (2014). The bisection point across variants of the task. *Attention, Perception, & Psychophysics*, *76*, 1671–1697. doi:10.3758/s13414-014-0672-9

Nash, J. C. (2014). On best practice optimization methods in R. *Journal of Statistical Software*, *60*(2), 1–14. doi:10.18637/jss.v060.i02

Ulrich, R., & Vorberg, D. (2009). Estimating the difference limen in 2AFC tasks: Pitfalls and improved estimators. *Attention, Perception, & Psychophysics*, *71*, 1219–1227. doi:10.3758/APP.71.6.1219
